# Gastric emptying in pregnancy and its clinical implications: a narrative review

**DOI:** 10.1016/j.bja.2024.09.005

**Published:** 2024-10-22

**Authors:** Jacob Lawson, Ryan Howle, Petar Popivanov, Jas Sidhu, Camilla Gordon, Maria Leong, Desire Onwochei, Neel Desai

**Affiliations:** 1 Department of Anaesthesia, Guy's and St Thomas' NHS Foundation Trust, London, UK; 2 Department of Anaesthesia, Rotunda Hospital, Dublin, Ireland; 3 Department of Anaesthesia, Coombe Hospital, Dublin, Ireland; 4 King's College London, London, UK

**Keywords:** fasting, gastric emptying, postpartum, pregnancy, ultrasound

## Abstract

Delayed gastric emptying increases the risk of pulmonary aspiration during anaesthesia for Caesarean delivery. Our aim in conducting this narrative review was to consider the effect of pregnancy on gastric emptying. The indices of gastric emptying after liquids, solids, or both and when fasted in the various trimesters of pregnancy, at the time of Caesarean delivery, in labour, and the postpartum period were assessed. We considered 32 observational studies, one nonrandomised controlled study, and 22 randomised controlled trials. The evidence indicates that, compared with the nonpregnant state, gastric emptying is decreased in the first but not the second and third trimesters. Before elective Caesarean delivery, carbohydrate drink or tea with milk leads to no difference in gastric cross-sectional area at 2 h relative to fasting or water. Following a standard fast for elective Caesarean delivery, patients may still have high-risk gastric contents. Compared with the nonpregnant state and third trimester, gastric emptying is delayed in labour, although the choice of analgesia has modifying effects. Systemic opioids delay gastric emptying. Epidural analgesia increases gastric emptying, but not back to baseline. Intrathecal analgesia delays gastric emptying relative to epidural analgesia. Women in labour who have eaten solids in the last 8 h still have high-risk gastric contents present in the stomach. The evidence with respect to the postpartum period is conflicting. In conclusion, inconsistencies in the literature reflect the unpredictability of gastric emptying in pregnancy and underline the potential value of gastric ultrasound in women who are pregnant.

**Editor's key points**

• Delayed gastric emptying increases the risk of pulmonary aspiration during anaesthesia for Caesarean delivery. Gastric emptying is decreased in the first but not the second and third trimesters.
• Before elective Caesarean delivery, carbohydrate drink or tea with milk results in no difference in gastric cross-sectional area compared with fasting or water. Gastric emptying is delayed in labour and with systemic opioids; it is increased by epidural analgesia, but not back to baseline.
• Inconsistencies in the literature reflect the unpredictability of gastric emptying in pregnancy.

In 1946, Mendelson described the association between the pulmonary aspiration of gastric contents and the occurrence of respiratory complications in 66 patients, most of whom delivered vaginally under ether and nitrous oxide anaesthesia.^1^ Cyanosis, dyspnoea, and tachycardia were noted in most women, but two died from airway obstruction due to solid and undigested food.^2^ The risk factors for pulmonary aspiration under anaesthesia encompass those related to the patient, the operation, and the anaesthesia.^3^^.^Risk factors associated with the patient include conditions leading to delayed gastric emptying, those related to the operation include length of surgery and patient position, and risk factors associated with the anaesthesia include gastric insufflation, difficult tracheal intubation, and inadequate depth of anaesthesia. Other risk factors for pulmonary aspiration have been identified in pregnancy such as decreased gastric pH, the effect of the gravid uterus on abdominal pressure, and progesterone-mediated relaxation of the lower oesophageal sphincter.^4^ Since the publication of Mendelson's findings, concerns have been raised in regard to the possibility of delayed gastric emptying in pregnancy.^5^ Delayed gastric emptying is a worry if loss of consciousness results from high neuraxial block with regional anaesthesia or when general anaesthesia is needed. These concerns could have contributed to the nonuniformity of national and international guidelines in regard to fasting before elective and emergent surgical procedures, such as cervical suture or Caesarean delivery, and in labour.^6^

In the case of Caesarean delivery, guidelines from the American Society of Anesthesiologists (ASA) and Society for Obstetric Anesthesia and Perinatology (SOAP) in the USA advocate a fasting period of 2 h for clear liquids and 6–8 h for solids.^7^ It is difficult to determine the exact timing of operations and patients are often kept fasted for a prolonged preoperative period, leading to adverse effects.^8^ Given this, global interest in the ‘Sip-Til-Send’ initiative has enabled them to drink sips of water only until they are called for to surgery. It has decreased the incidence of nausea and vomiting, light headedness, and thirst, increased comfort, and reduced the rate of anxiety in women.^9^ Further, in the United Kingdom, a single institution has reported no occurrences of pulmonary aspiration following the use of unrestrictive sips of water in women before over 2000 elective Caesarean deliveries.^10^ In the setting of labour, recommendations from the ASA and SOAP suggest moderate clear liquids in patients who do not have risk factors for pulmonary aspiration or operative delivery and the avoidance of food.^7^ Similarly, guidance from the European Society of Anaesthesiology (ESA) advocates clear liquids and the discouragement of food.^11^ The National Institute for Health and Care Excellence (NICE) in the United Kingdom, however, recommend that women may eat a light diet in labour unless they have developed risk factors that increase the likelihood of Caesarean delivery or received opioid.^12^ Importantly, in a previous survey, the restriction of oral liquids and solids resulted in moderate or high stress in 57% and 27% of patients, respectively,^13^ and in a recent study, 12% of women stated that they would have liked to eat some solids in labour.^14^

Studies that evaluate gastric emptying use different methods such as gastric ultrasound, paracetamol absorption, or scintigraphy.^15^^,^^16^ There is a clear requirement for a comprehensive review of the evidence base and literature in order to elucidate how gastric emptying changes in the different phases of pregnancy, if at all, compared with patients who are not pregnant. No review, to the knowledge of the authors, has been published to date with respect to gastric emptying that incorporates all the results of observational studies and randomised controlled trials.

In view of this, our aim was to perform a narrative review to evaluate the indices of gastric emptying in response to liquids, solids, or both and when fasted in the various trimesters of pregnancy, at the time of Caesarean delivery, in labour, and the postpartum period, and relative to women who are not pregnant. Moreover, we sought to review the influence of anaesthetic-related medications such as epidural local anaesthetic with or without opioid on these markers of gastric emptying and the clinical implications of the gastric emptying in these phases of pregnancy on clinical practice.

## Methods

In this narrative review, our inclusion criteria consisted of females who were pregnant or in the postpartum period and had an evaluation of their gastric emptying performed while fasted or in response to liquids, solids, or both. This included women who were in any trimester of pregnancy, in labour, or having elective or emergency Caesarean delivery. Gastric emptying in the different phases of pregnancy or the postpartum period may have been compared or it may have been compared relative to the nonpregnant phase. The exclusion criteria involved any single and standalone assessment of gastric content or volume without ongoing evaluation of the change in these indices over time. Using a mixed methods approach, we included observational studies, nonrandomised controlled trials, and randomised controlled trials. No restrictions were made on the language of publication.

We conducted a search strategy from inception to January 24, 2024 of the following databases: Cochrane Central Register of Controlled Trials (CENTRAL), Cumulative Index to Nursing and Allied Health Literature (CINAHL), Elton B Stephens Company (EBSCO) Global Health, Excerpta Medica (Embase), Medical Literature Analysis and Retrieval System Online (MEDLINE), Scopus, and World of Science (WoS). The full search included free text keywords and medical subject headings such as gastric emptying, gastric motility, stomach, obstetric, and pregnancy (Supplementary Appendix S1). Subsequent to deduplication, all citations were uploaded to Rayyan, the reference managing software (Qatar Computing Research Institute, 2016, Doha, Qatar),^17^ and abstract screening of these citations was performed by two blinded authors (RH, PP). Discrepancies were resolved through discussion or, if no agreement was reached, by a third author (ND). The full text of all potentially eligible citations was retrieved before a final screening for eligibility by one author (ND), and a single author (CG) hand searched the reference lists of included studies for additional citations relevant to this review.

The following data, if available, were extracted by authors (JL, RH, ND, JS, CG, PP) from the included studies: design of study; trimester of pregnancy or time since delivery; labour or not; Caesarean delivery or not; comparator groups; number of patients; method of assessing gastric emptying and conditions related to this evaluation such as whether it was performed subsequent to drug administration or ingestion of solids; and relevant findings such as the gastric half emptying time. The methodological quality and risk of bias of studies were examined by two blinded authors (ML, DO), with discrepancies resolved via discussion or, if required, a third author (ND). For nonrandomised studies and randomised controlled trials, the Risk of Bias In Non-randomised Studies – of Interventions (ROBINS-I) and the revised Cochrane Risk of Bias 2 (RoB 2) tool were used, respectively.^18^^,^^19^

## Results

**Fig 1:**
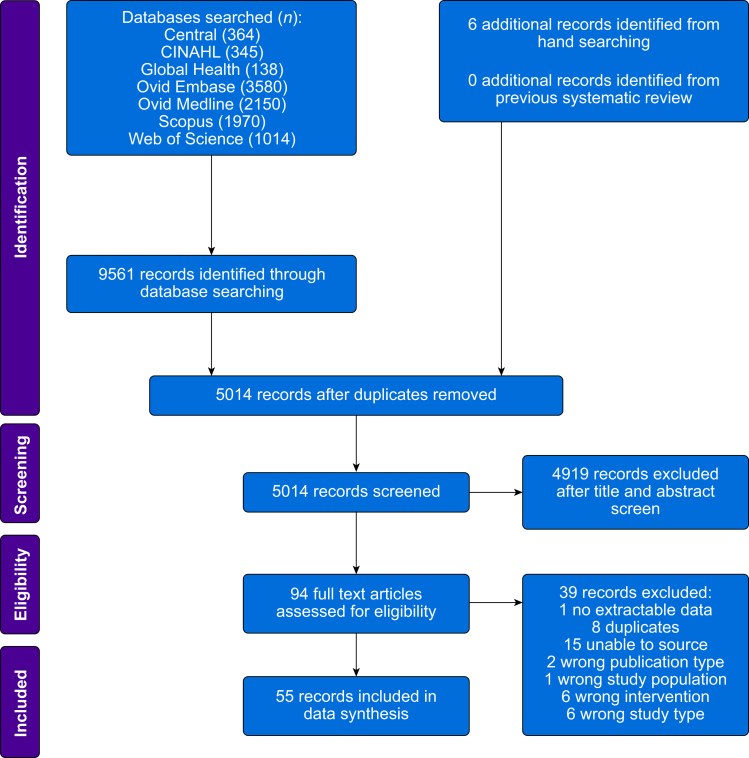
PRISMA flow diagram summarising the retrieved, included, and excluded studies. PRISMA, Preferred Reporting Items for Systematic Reviews and Meta-Analyses.

In all, 55 citations fulfilled the inclusion criteria (Fig. 1),^5^^,^^20^, ^21^, ^22^, ^23^, ^24^, ^25^, ^26^, ^27^, ^28^, ^29^, ^30^, ^31^, ^32^, ^33^, ^34^, ^35^, ^36^, ^37^, ^38^, ^39^, ^40^, ^41^, ^42^, ^43^, ^44^, ^45^, ^46^, ^47^, ^48^, ^49^, ^50^, ^51^, ^52^, ^53^, ^54^, ^55^, ^56^, ^57^, ^58^, ^59^, ^60^, ^61^, ^62^, ^63^, ^64^, ^65^, ^66^, ^67^, ^68^, ^69^, ^70^, ^71^, ^72^, ^73^ and 32 were observational studies,^5^^,^^20^^,^^21^^,^^24^, ^25^, ^26^, ^27^, ^28^, ^29^, ^30^, ^31^, ^32^, ^33^^,^^35^^,^^38^^,^^40^^,^^42^^,^^51^^,^^57^, ^58^, ^59^, ^60^^,^^63^, ^64^, ^65^, ^66^^,^^68^, ^69^, ^70^, ^71^, ^72^, ^73^ one was a nonrandomised controlled study,^23^ and 22 were randomised controlled trials.^22^^,^^34^^,^^36^^,^^37^^,^^39^^,^^41^^,^^43^, ^44^, ^45^, ^46^, ^47^, ^48^, ^49^, ^50^^,^^52^, ^53^, ^54^, ^55^, ^56^^,^^61^^,^^62^^,^^67^

The studies were composed of five different patient cohorts: nonpregnant (18)^5^^,^^21^, ^22^, ^23^, ^24^, ^25^, ^26^, ^27^, ^28^, ^29^^,^^64^^,^^66^, ^67^, ^68^, ^69^, ^70^, ^71^^,^^73^; first trimester (T1), defined as start of pregnancy to week 12 (10)^21^^,^^22^^,^^24^^,^^26^, ^27^, ^28^, ^29^, ^30^^,^^32^^,^^72^; second trimester (T2), that is weeks 13–26 of pregnancy (5)^21^^,^^26^^,^^28^^,^^30^^,^^72^; third trimester (T3), that is week 27 of pregnancy until labour (19)^2^^,^^5^^,^^20^^,^^21^^,^^23^^,^^25^^,^^26^^,^^28^^,^^30^, ^31^, ^32^^,^^38^^,^^53^^,^^64^^,^^65^^,^^70^, ^71^, ^72^, ^73^; Caesarean delivery (8)^36^^,^^51^^,^^52^^,^^54^, ^55^, ^56^, ^57^, ^58^; labour (20)^33^, ^34^, ^35^, ^36^, ^37^^,^^39^, ^40^, ^41^^,^^43^, ^44^, ^45^, ^46^, ^47^, ^48^, ^49^, ^50^^,^^59^^,^^64^^,^^70^^,^^71^; postpartum, that is from immediately following delivery until 6 weeks thereafter (15)^21^^,^^35^^,^^57^^,^^59^, ^60^, ^61^, ^62^, ^63^^,^^65^, ^66^, ^67^, ^68^, ^69^^,^^72^^,^^73^; and nonpregnant after postpartum, defined as greater than 6 weeks but less than 15 months subsequent to delivery due to the potential of residual physiological changes from pregnancy and ongoing breast feeding (3).^28^^,^^30^^,^^32^

**Fig 2:**
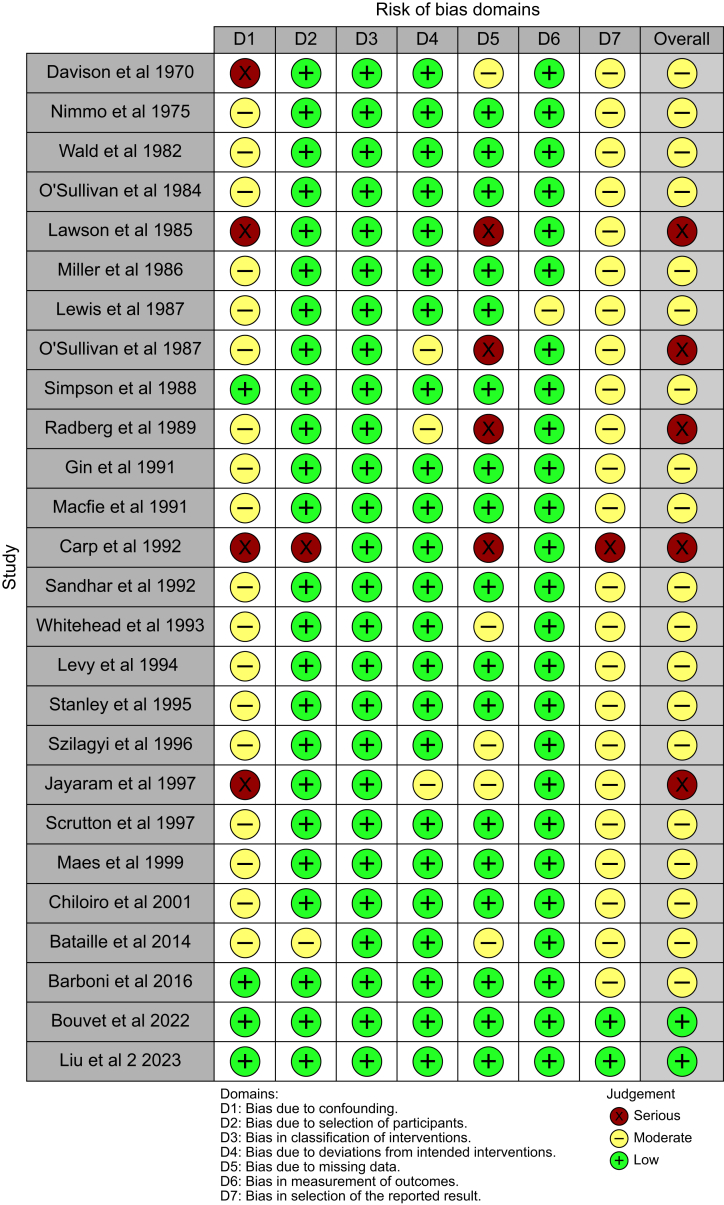
Risk of bias assessment of the observational studies using the ROBINS-I tool.^5^^,^^21^^,^^23^^,^^24^, ^25^, ^26^, ^27^, ^28^, ^29^, ^30^^,^^32^^,^^35^^,^^38^^,^^54^^,^^58^^,^^60^^,^^63^, ^64^, ^65^, ^66^^,^^68^, ^69^, ^70^, ^71^, ^72^, ^73^ +, low risk; !, moderate risk; –, serious risk.

**Fig 3:**
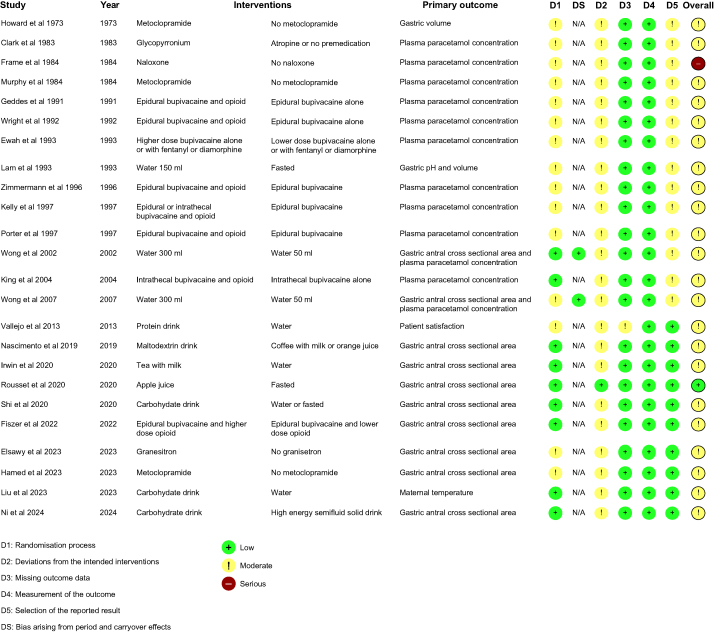
Risk of bias assessment of the randomised controlled trials using the ROB 2 tool. +, low risk; !, some concerns; –, high risk.

Of the observational studies that were assessed with ROBINS-I, just two were demonstrated to have low risk of bias^54^^,^^71^ and the remaining 19 and five were found to be at moderate^5^^,^^21^^,^^23^^,^^24^^,^^26^, ^27^, ^28^, ^29^, ^30^^,^^32^^,^^35^^,^^38^^,^^58^^,^^60^^,^^63^, ^64^, ^65^, ^66^^,^^69^ or high^25^^,^^68^^,^^70^^,^^72^^,^^73^ risk of bias, respectively (Fig. 2). Deficiencies in the observational studies were mainly shown in the domains of confounding, missing data and selection of the reported result. Of the randomised controlled trials that were evaluated with RoB2, only one was demonstrated to have low risk of bias,^39^ and the remaining 22 and one were found to be at moderate^20^^,^^22^^,^^31^^,^^34^^,^^36^^,^^37^^,^^40^^,^^41^^,^^43^, ^44^, ^45^, ^46^, ^47^, ^48^, ^49^^,^^52^^,^^53^^,^^55^^,^^56^^,^^61^^,^^62^^,^^67^ or high^50^ risk of bias, respectively (Fig. 3). Problems in the randomised controlled trials were predominantly found in the domains of randomisation process, deviations from the intended interventions, and selection of the reported result.

The characteristics and results of the studies have been detailed in Table 1. Ten different methods were used to objectively assess gastric emptying: gastric ultrasound (24)^5^^,^^20^^,^^25^^,^^31^^,^^32^^,^^34^^,^^37^, ^38^, ^39^, ^40^^,^^42^^,^^47^^,^^48^^,^^51^, ^52^, ^53^, ^54^, ^55^, ^56^^,^^59^^,^^68^, ^69^, ^70^, ^71^; paracetamol absorption (20)^20^, ^21^, ^22^^,^^24^^,^^26^^,^^27^^,^^30^^,^^31^^,^^35^^,^^36^^,^^41^^,^^43^, ^44^, ^45^, ^46^^,^^50^^,^^60^, ^61^, ^62^^,^^66^; nasogastric sampling with (2)^49^^,^^64^ and without dye dilution (3)^25^^,^^58^^,^^67^; breath hydrogen analysis (4)^28^^,^^32^^,^^63^^,^^72^; epigastric impedance (2)^65^^,^^73^; barium X-ray (1)^33^; breath ^13^C-octanoic acid analysis (1)^29^; electrogastrography (1)^57^; and radiotelemetry pH pill (1).^23^ Five studies utilised more than one of these methods^20^^,^^25^^,^^31^^,^^32^^,^^69^ and one investigated gastric emptying by using gastric ultrasound with scintigraphy.^69^ Studies varied in relation to the nature of what was consumed by the patient to evaluate gastric emptying. In 24 studies, they had drugs and liquids,^20^, ^21^, ^22^, ^23^, ^24^^,^^26^, ^27^, ^28^^,^^31^^,^^32^^,^^35^^,^^36^^,^^41^^,^^43^^,^^45^^,^^46^^,^^50^^,^^60^, ^61^, ^62^, ^63^^,^^65^^,^^66^^,^^72^ in 17, liquids alone,^25^^,^^33^^,^^34^^,^^37^, ^38^, ^39^^,^^42^^,^^44^^,^^49^^,^^51^, ^52^, ^53^, ^54^^,^^59^^,^^64^^,^^67^^,^^73^ in eight, liquids and solids,^5^^,^^30^^,^^32^^,^^40^^,^^47^^,^^48^^,^^58^^,^^70^ in three, solids alone,^29^^,^^68^^,^^71^ in two, drugs, liquids, and solids,^30^^,^^69^ and in one, no drugs, liquids or solids.^57^

**Table 1:** Characteristics of the included trials. All Caesarean deliveries were elective unless otherwise stated. Differences only reported as significant if *P*<0.05. CSA, cross-sectional area; OCTT, orocaecal transit time; RLSR, right lateral semirecumbent; T1, first trimester; T2, second trimester; T3, third trimester; US, ultrasound.

Reference	Group (*n*)	Type of publication	Constituents of groups	Methodology	Drug, liquids, solids, or both	Conditions or interventions if applicable	Relevant findings
**Pregnancy**							
Wong *et al.,* 2002^20^	Pregnant (T3) (11)	Cross-over study	BMI <35 kg m^−2^	Gastric US (9) and paracetamol absorption (11)	Drug and liquids	Following an overnight fast, ingestion of paracetamol 1.5 g with water 50 or 300 ml on two occasions separated by at least 2 days Blood sampling then performed every 10–30 min for 1–2.5 h and gastric US in semirecumbent position at 45 degrees every 10 min for 1 h or until stomach empty	Gastric half emptying time decreased with water 300 ml (24 [sd 6] min) compared with 50 ml (33 [8] min) Time to peak plasma paracetamol concentration reduced with water 300 ml (40.9 [19.2] min) compared with 50 ml (24.6 [12.1] min) No difference in area under plasma paracetamol curve at 1 h or peak plasma paracetamol concentration between water 50 and 300 ml
Wong *et al.,* 2007^31^	Pregnant (T3) (10)	Cross-over study	BMI >35 kg m^−2^	Gastric US and paracetamol absorption	Drug and liquids	Following an overnight fast, ingestion of paracetamol 1.5 g with water 50 or 300 ml on two occasions separated by at least 2 days Blood sampling then performed every 10–30 min for 1–2 h and gastric US in semirecumbent position at 45 degrees every 10 min for 1 h or until stomach empty	No difference in area under plasma paracetamol curve, peak plasma paracetamol concentration or time to peak plasma paracetamol concentration between water 50 and 300 ml
Sutanto *et al.,* 2012^42^	Pregnant (T3) (9)	Cross-sectional study	–	Gastric US	Liquids	Following a fast of 2 h for clear liquids and 6 h for solids, ingestion of liquid formula 200 ml (carbohydrate 62%, fat 20%, and protein 18% with total of 200 kcal) Gastric US then performed in an unspecified position every 30 min for 2 h	Preingestion gastric volume averaged 41.8 [18.7] ml All patients had a gastric volume of <80 ml in <2 h
Irwin *et al.,* 2020^53^	Pregnant (T3) (50)	Randomised controlled trial	Tea with milk (25) Water (25)	Gastric US	Liquids	Following a fast of at least 2 h for clear fluids, 6 h for light meal and 8 h for fatty meal, ingestion of tea with milk 200 ml or water 250 ml Gastric US then performed in RLSR and semirecumbent position at 45 degrees every 15 min for 2 h	No difference in gastric antral CSA and total gastric fluid volume at 2 h between tea with milk and water
Davison *et al.,* 1970^64^	Pregnant (T3) (11) Labour (8) Nonpregnant (11)	Cross-sectional study	–	Nasogastric sampling and dye dilution	Liquids	Following gastric content aspiration, ingestion of water 750 ml that contained phenol red 30 μg ml^−1^ 1 h later Nasogastric sampling then performed every 10 min	No difference in gastric half emptying time or volume remaining in stomach at 30 min between T3 and nonpregnant
Carp *et al.,* 1992^70^	Pregnant (T3) (34) Labour (39) Nonpregnant (20)	Cross-sectional study	Pregnant: Standard meal (20) Unspecified solid food (14) Nonpregnant: Standard meal (20)	Gastric US	Liquids and solids	Following an overnight fast, ingestion of a standard meal 800 g Gastric US then performed in RLSR at 45 degrees or semirecumbent position every 2 h Following an unspecified fast, ingestion of unspecified solid food Gastric US in an unspecified position then performed once at varying time intervals	No food detected in stomach at 4 h in T3 and nonpregnant
Bouvet *et al.,* 2022^71^	Pregnant (T3) (10) Labour (20) Nonpregnant (10)	Cross-sectional study	–	Gastric US	Solids	Following a fast of at least 1 h for clear fluids and 6 h for solids, ingestion of a standard meal (carbohydrate 21.2 g, fat 2.2 g and protein 3.8 g with a total of 120 kcal) Gastric US then performed in semirecumbent position at 45 degrees at 15 min and 1, 1.5, and 2 h	No difference in gastric half emptying time between T3 and nonpregnant
Lawson *et al.,* 1985^72^	Pregnant (T1–3) (42) Postpartum (17)	Prospective cohort study	Pregnant: T1 (8) T2 (12) T3 (22)	Breath hydrogen analysis	Drug and liquids	Following an overnight fast, ingestion of lactulose 10 g with water 100 ml Breath sampling then performed every 15 min for 4 h	OCTT increased with progression of pregnancy (T1 99 [39] *vs* T2 125 [48] *vs* T3 137 [58] min)
O'Sullivan *et al.,* 1987^73^	Pregnant (T3) (18) Postpartum (15) Nonpregnant (13)	Cross-sectional study	–	Epigastric impedance	Liquids	Following a fast of at least 4 h, ingestion of water or diluted orange concentrate 500 ml Electrical impedance then measured through electrodes on the epigastrium	No difference in gastric half emptying time between T3 and nonpregnant
Whitehead *et al.,* 1993^21^	Pregnant (T1–3) (64) Postpartum (55) Nonpregnant (32)	Prospective cohort study	Pregnant: T1 (18) T2 (10) T3 (36)	Paracetamol absorption	Drug and liquids	Following a fast of least 4 h, ingestion of paracetamol 1.5 g with water 50 ml Blood sampling then performed every 10–30 min for 2 h	No difference in area under plasma paracetamol curve at 2 h, peak plasma paracetamol concentration, or time to peak plasma paracetamol concentration between T1, T2, T3, and nonpregnant
Clark *et al.,* 1983^22^	Pregnant (T1) (30) Nonpregnant (30)	Randomised controlled trial	Pregnant and nonpregnant: No premedication (10) Atropine 300 μg (10) Glycopyrrolate 600 μg (10)	Paracetamol absorption	Drug and liquids	Following a fast of at least 6 h, ingestion of paracetamol 500 mg with water 12.5 ml Blood sampling then performed every 15–30 min for 2 h	In the presence of no premedication, area under plasma paracetamol curve at 1 h decreased in T1 (44.7 [12.2] μg ml^−1^ h^−1^) compared with nonpregnant (53.7 [16.5] μg ml^−1^ h^−1^) In the context of pregnancy, area under plasma paracetamol curve at 1 h reduced by glycopyrrolate (20.7 [10.7] μg ml^−1^ h^−1^) compared with no premedication (44.7 [12.2] μg ml^−1^ h^−1^) and atropine (35.8 [14.2] μg ml^−1^ h^−1^)
O'Sullivan *et al.,* 1984^23^	Pregnant (T3) (23) Nonpregnant (11)	Nonrandomised controlled study	Pregnant: Magnesium trisilicate (12) Sodium citrate (11) Nonpregnant: Sodium citrate (11)	Radiotelemetry pH pill	Drug and liquids	Following an overnight fast, ingestion of pH pill with water 15–20 ml and subsequent antacid 15 ml Continuous pH then measured while within the stomach	No difference in gastric half emptying time or pH pill transit time between T3 and nonpregnant with the same antacid No difference in gastric half emptying time between sodium citrate and magnesium trisilicate in T3
Simpson *et al.,* 1988^24^	Pregnant (T1) (28) Nonpregnant (14)	Cross-sectional study	Pregnant: 8–11 weeks' gestation (16) 12–14 weeks' gestation (12)	Paracetamol absorption	Drug and liquids	Following a fast of least 4 h, ingestion of paracetamol 1.5 g with water 150 ml Blood sampling then performed every 15–30 min for 2 h	Area under plasma paracetamol curve at 1 h decreased in 12–14 weeks' pregnant (10.5 [5.9] μg ml^−1^ h^−1^) compared with nonpregnant (19.3 [7.1] μg ml^−1^ h^−1^) Peak plasma paracetamol concentration reduced in 12–14 weeks' pregnant (21.4 [7.6] μg ml^−1^) compared with nonpregnant (34.4 [16.1] μg ml^−1^) Time to peak plasma paracetamol concentration increased in 12–14 weeks' pregnant (71.9 [31.9] min) compared with nonpregnant (45 [22.1] min)
Rådberg *et al.,* 1989^25^	Pregnant (T3) (14) Nonpregnant (16)	Cross-sectional study	Nasogastric sampling: Pregnant (5) Nonpregnant (7)	Gastric US and nasogastric sampling	Liquids	Following an overnight fast, ingestion of liquid formula 300 ml (carbohydrate 17 g, fat 5 g, and protein 5 g) Gastric US then performed in erect position using the sum of cylinders method every 15 min for 1.75 h before nasogastric sampling in some patients for nitrogen analysis	No difference in final gastric volume compared with the starting volume or residual gastric protein content at 1.75 h between T3 and nonpregnant
Macfie *et al.,* 1991^26^	Pregnant (T1–3) (45) Nonpregnant (15)	Cross-sectional study	Pregnant: T1 (15) T2 (15) T3 (15)	Paracetamol absorption	Drug and liquids	Following a fast of at least 6 h, ingestion of paracetamol 1.5 g with water 50 ml Blood sampling then performed every 15–30 min for 2 h	Area under plasma paracetamol curve at 1 h decreased in T1 (8.8 [1.5] μg ml^−1^ h^−1^) compared with nonpregnant (14 [1.5] μg ml^−1^ h^−1^) No difference in area under plasma paracetamol curve at 1 h between T2 or T3 and nonpregnant No difference in peak plasma paracetamol concentration or time to peak plasma paracetamol concentration between T1, T2, T3 and nonpregnant
Levy *et al.,* 1994^27^	Pregnant (T1) (20) Nonpregnant (20)	Cross-sectional study	–	Paracetamol absorption	Drug and liquids	Following a fast of at least 4 h, ingestion of paracetamol 1.5 g with water 50 ml Blood sampling then performed every 15 min for 2 h	Area under plasma paracetamol curve at 1 h decreased in T1 (10.6 [5.5] μg ml^−1^ h^−1^) compared with nonpregnant (17.9 [8.9] μg ml^−1^ h^−1^) Peak plasma paracetamol concentration reduced in T1 (3.3 [7.5] μg ml^−1^) compared with nonpregnant (29.9 [11.5] μg ml^−1^) Time to peak plasma paracetamol concentration increased in T1 (69.0 [29.0] min) compared with nonpregnant (48.0 [28.2] min)
Szilagyi *et al.,* 1996^28^	Pregnant (T1–2) (30) Nonpregnant after postpartum (27) Nonpregnant (17)	Prospective cohort study	Nonpregnant after postpartum: 3–14 months following neonatal delivery	Breath hydrogen analysis	Drug and liquids	Following an overnight fast, ingestion of lactulose 10 g with water 100 ml Breath sampling then performed every 10 min for 3 h	OCTT increased in pregnant (99.2 [7.8] min) compared with nonpregnant after postpartum (68.5 [6.4] min) and nonpregnant (63.5 [8.7] min)
Maes *et al.,* 1999^29^	Pregnant (T1) (24) Nonpregnant (43)	Case control study	Pregnant: No previous hyperemesis gravidarum (10) Recovered hyperemesis gravidarum (14)	Breath ^13^C-octanoic acid analysis	Solids	Following an overnight fast, ingestion of a standard meal 60 g (containing ^13^C-octanoic acid 91 mg and total of 250 kcal) with water 150 ml Breath sampling then performed every 15 min for 4 h	Gastric half emptying time decreased in T1 recovered from hyperemesis gravidarum (62 [20.7] min) compared with T1 without previous hyperemesis (94 [21.5] min) No difference in gastric half emptying time between T1 recovered from hyperemesis gravidarum, T1 without previous hyperemesis and nonpregnant
Barboni *et al.,* 2016^5^	Pregnant (T3) (10) Nonpregnant (10)	Case control study	–	Gastric US	Liquids and solids	Following a fast of at least 6 h, ingestion of a standard meal (carbohydrate 55 g, fat 10 g, and protein 35 g with a total of 450 kcal) with water 300 ml Gastric US then performed in RLSR at 45 degrees at 10 min and 1.5 and 4 h	Change in gastric antral CSA at 10–90 min was decreased in T3 (–270 [320] mm^2^) compared with nonpregnant (–500 [640] mm^2^) Change in gastric antral CSA at 1.5–4 h was reduced in T3 (–440 [300] mm^2^) compared with nonpregnant (–500 [690] mm^2^)
Stanley *et al.,* 1995^30^	Pregnant (T1–3) (29) Nonpregnant after postpartum (10)	Prospective cohort study	Pregnant: T1 (10) T2 (9) T3 (10) Nonpregnant after postpartum: 8 weeks following neonatal delivery	Paracetamol absorption	Drug, liquids and solids	Following an 8-h fast, ingestion of a standard meal (carbohydrate 59 g, fat 21.2 g, and protein 17.9 g) with water 200 ml and subsequent paracetamol 1.5 g with water 50 ml Blood sampling then performed every 20 min for 2.7 h	No difference in peak plasma paracetamol concentration or time to peak plasma paracetamol concentration between T1, T2, T3, and nonpregnant after postpartum
Chiloiro *et al.,* 2001^32^	Pregnant (T1 and 3) (11) Nonpregnant after postpartum (11)	Prospective cohort study	Nonpregnant after postpartum: 4–6 months following neonatal delivery	Breath hydrogen analysis and gastric US	Drug and liquids	Following an overnight fast, ingestion of lactulose 18 g with water 180 ml Breath sampling then performed every 10 min for up to 6 h and gastric US in erect position every 10 min until stomach empty	OCTT increased in T3 (100 [95% IQR 50.5–240] min) compared with nonpregnant after postpartum (70 [95% IQR 0.5–240] min) No difference in gastric emptying between T1, T3, and nonpregnant after postpartum
**Caesarean delivery**							
*Carbohydrate drink*							
Popivanov *et al.,* 2020^51^	Caesarean delivery (40)	Prospective cohort study	–	Gastric US	Liquids	Following a fast of 2 h for clear fluids, 6 h for light meal, and 8 h for fatty meal or meat, ingestion of carbohydrate drink 400 ml (carbohydrate 40 g) up to 2 h before Caesarean delivery Gastric US then performed in RLSR at 45 degrees and supine position before ingestion and every 20 min for 2 h subsequent to ingestion	No solid food detected in gastric antrum and Perlas grade was 0 and 1 in 80% and 20% of patients, respectively, before ingestion Perlas grade at 1.7 h was 0 and 1 in 77.5% and 22.5% of patients, respectively, following ingestion No difference in gastric antral CSA before ingestion and subsequent to ingestion at 2 h
Shi *et al.,* 2021^52^	Caesarean delivery (75)	Randomised controlled trial	Fasted (25) Carbohydrate drink (25) Water (25)	Gastric US	Liquids	Following a 6-h fast, fasting continued for 2 h or ingestion of carbohydrate drink 300 ml or water 300 ml 2 h before Caesarean delivery Gastric US then performed in supine position before ingestion, subsequent to ingestion, and 2 h thereafter	No difference in gastric antral CSA at baseline and 2 h between carbohydrate drink and water
Liu *et al.,* 2023^54^	Caesarean delivery (90)	Randomised controlled trial	Water (45) Carbohydrate drink (45)	Gastric US	Liquids	Following a fast of at least 2 h for clear fluids, 6 h for light meal, and 8 h for fatty meal, combined spinal epidural with intrathecal isobaric ropivacaine 0.5%, 3 ml and, if needed, epidural lidocaine 2% for Caesarean delivery Subsequent to arrival to recovery, ingestion of carbohydrate drink 100 ml (carbohydrate 10 g) or water 10 ml Gastric US then performed in RLSR at 45 degrees before ingestion and at 5 and 30 min and 1, 1.5, and 2 h subsequent to ingestion Gastric fullness if gastric antral CSA >1030 mm^2^	Gastric antral CSA increased at 30 min with carbohydrate drink (1070 [270] mm^2^) compared with water (780 [150] mm^2^) after Caesarean delivery Gastric antral CSA increased at 1 h with carbohydrate drink (810 [200] mm^2^) compared with water (620 [130] mm^2^) following Caesarean delivery No difference in gastric antral CSA at 1.5 and 2 h between water and carbohydrate subsequent to Caesarean delivery 13.3%, 6.7%, and 2.2% of patients had gastric fullness at 1, 1.5, and 2 h, respectively
*Miscellaneous drugs*							
Murphy *et al.,* 1984^36^	Labour (80) Caesarean delivery (40)	Randomised controlled trial	Caesarean delivery: No metoclopramide (20) Metoclopramide (20)	Paracetamol absorption	Drug and liquids	Patients in Caesarean delivery group received aluminium hydroxide 30 ml before induction of unspecified anaesthesia Following a fast of at least 2 h, ingestion of paracetamol 1.5 g with water 200 ml Blood sampling then performed at 1 h	Plasma paracetamol concentration at 1 h decreased in Caesarean delivery without metoclopramide (13.6 [6.5] μg ml^−1^) compared with Caesarean delivery with metoclopramide (17.9 [4.7] μg ml^−1^
Elsawy *et al.,* 2023^55^	Caesarean delivery (60)	Randomised controlled trial	No granisetron (30) Granisteron (30)	Gastric US	Drug	Following a 6–8-h fast, gastric US performed in RLSR at 45 degrees 1 h before Caesarean delivery Subsequent to no granisetron or granisetron, gastric US then repeated in RLSR at 45 degrees 1 h later	Gastric antral CSA decreased at 1 h (400 [120] mm^2^) with granisetron but not no granisetron compared with baseline (480 [140] mm^2^) Gastric volume reduced at 1 h (50 [19] ml) with granisetron but not no granisetron compared with baseline (70 [24] ml)
Hamed *et al.,* 2023^56^	Caesarean delivery (111)	Randomised controlled trial	No metoclopramide (55) Metoclopramide (56)	Gastric US	Drug	Following a 7-h fast, gastric US performed in RLSR and supine position at 45 degrees 1 h before Caesarean delivery Subsequent to no metoclopramide or metoclopramide, gastric US then repeated in RLSR and supine position at 45 degrees 1 h later	Gastric antral CSA decreased at 1 h (520 [120] mm^2^) with metoclopramide but not no metoclopramide compared with baseline (760 [130] mm^2^) Gastric volume reduced at 1 h (69 [19] ml) with metoclopramide but not no metoclopramide compared with baseline (104 [19] ml) Perlas grade was 2 in 7.1% of patients who had metoclopramide compared with 46.2% of patients who had not had metoclopramide
*Regional anaesthesia*							
Oshima *et al.,* 2009^57^	Caesarean delivery (16) Postpartum (16)	Prospective cohort study	–	Electrogastrography	None	Following an overnight fast, combined spinal epidural with intrathecal hyperbaric bupivacaine 0.5%, 2 ml and fentanyl 10 μg and, if needed, epidural ropivacaine 0.375% for Caesarean delivery Electrogastrography then performed 10 min before and 10 and 20 min subsequent to combined spinal epidural, 10 min before end of Caesarean delivery and on postoperative day 7	Dominant frequency of electrogastrography at 10 min and 20 min following combined spinal epidural and 10 min before end of Caesarean delivery increased compared with baseline Electrogastrography at postoperative day 7 increased compared with baseline, 10 min, and 20 min subsequent to combined spinal epidural and 10 min before end of Caesarean delivery
*General anaesthesia*							
Lewis *et al.,* 1987^58^	Caesarean delivery (40)	Prospective cohort study	Fasted (20) Liquids (9) Liquids and solids (11)	Nasogastric sampling	Liquids and solids	Following an overnight fast, ingestion of magnesium trisilicate 20 ml Patients then given no liquids or solids, tea, or tea and toast up to 4 h before Caesarean delivery General anaesthesia with thiopentone 5 mg kg^−1^ and suxamethonium 100 mg for Caesarean delivery Subsequent to neonatal delivery, they received fentanyl 200 μg and nasogastric aspiration then performed	Gastric aspirate volume with liquids (73.4 [52.7] ml) or liquids and solids (64.5 [21.5] ml) increased compared with fasted (33.2 [22] ml)
**Labour**							
*Labour in absence of or without specified epidural*							
Hirsheimer *et al.,* 1983^33^	Labour (10)	Cross-sectional study	Unspecified analgesia	Barium X-ray	Liquids	Following an unspecified fast, ingestion of barium 170 ml X-ray then performed every h for 4 h	Gastric emptying delayed in 20% of patients based on barium distribution on serial X-rays
Nascimento *et al.,* 2019^34^	Labour (54)	Randomised controlled trial	Maltodextrin (18) Coffee with milk (18) Orange juice (18) Unspecified analgesia	Gastric US	Liquids	Following a fast of least 2 h for clear fluids, 4 h for light meal and 6–8 h for high fat or protein meal, ingestion of coffee with milk, maltodextrin or orange juice, all 450 ml and with a total of 200 kcal Gastric US performed in RLSR at 45 degrees before ingestion and then at 5 min and every 30 min for 2 h	Gastric emptying of coffee with milk and orange juice decreased compared with maltodextrin in labour No difference in gastric emptying between coffee with milk and orange juice No patient who received maltodextrin had an estimated gastric volume >1.5 ml kg^−1^ at 1.5 h or a gastric antral CSA greater at 1.5 h compared with baseline in labour
Davison *et al.,* 1970^64^	Pregnant (T3) (11) Labour (8) Nonpregnant (11)	Cross-sectional study	Labour: Unspecified analgesia	Nasogastric sampling and dye dilution	Liquids	Following gastric content aspiration, ingestion of water 750 ml containing phenol red 30 μg ml^−1^ 1 h later Nasogastric sampling then performed every 10 min	Gastric volume at 30 min increased in labour (393 [126.1] ml) compared with T3 (274.8 [115.1] ml) and nonpregnant (186 [96.5] ml)
*Influence of different analgesic strategies excluding epidural*							
Nimmo *et al.,* 1975^35^	Labour (46) Postpartum (10)	Cross-sectional study	Labour: No opioid (12) IM diamorphine 10 mg (8) IM diamorphine 10 mg and metoclopramide 10 mg (5) I.M. pentazocine 60 mg (8) I.M. meperidine 150 mg (8) I.M. meperidine 150 mg and metoclopramide 10 mg (5)	Paracetamol absorption	Drug and liquids	Following a fast of at least 4 h and a variable period following opioid, ingestion of paracetamol 1.5 g with water 200 ml Blood sampling then performed every 15 min for 1.5 h and subsequently every 1–2 h up to 8 h	Plasma paracetamol concentration decreased at 1 h with pentazocine in labour (2 [1.5] μg) compared with no opioid in labour (18.7 [4.6] μg) Peak plasma paracetamol concentration reduced with meperidine (14 [8.3] μg ml^−1^) and diamorphine in labour (8.6 [4.6] μg ml^−1^) compared with no opioid in labour (21 [10.3] μg ml^−1^) Time to peak plasma paracetamol increased with meperidine (4 h) and diamorphine in labour (4 h) compared with no opioid in labour (30 min) Inhibitory effect of diamorphine and meperidine on gastric emptying in labour not reversed by metoclopramide
Murphy *et al.,* 1984^36^	Labour (80) Caesarean delivery (40)	Randomised controlled trial	Labour without opioid: No metoclopramide (20) Metoclopramide (20) Labour with opioid: No metoclopramide (20) Metoclopramide (20) Caesarean delivery: No metoclopramide (20) Metoclopramide (20)	Paracetamol absorption	Drug and liquids	Patients in labour opioid group given i.m. meperidine 50 mg Those in caesarean delivery group received aluminium hydroxide 30 ml before induction of unspecified anaesthesia Following a fast of at least 2 h, ingestion of paracetamol 1.5 g with water 200 ml Blood sampling then performed at 1 h	Plasma paracetamol concentration at 1 h decreased in labour with opioid (6.4 [8.4] μg ml^−1^) compared with labour without opioid (9.4 [6.6] μg ml^−1^), and reduced in labour without opioid (9.4 [6.6] μg ml^−1^) relative to Caesarean delivery (13.6 [6.5] μg ml^−1^) Plasma paracetamol concentration at 1 h decreased in Caesarean delivery without metoclopramide (13.6 [6.5] μg ml^−1^) compared with Caesarean delivery with metoclopramide (17.9 [4.7] μg ml^−1^), reduced in labour without metoclopramide (9.4 [6.5] μg ml^−1^ relative to labour with metoclopramide (15.9 [5.6] μg ml^−1^), and decreased in labour with opioid (6.4 [8.4] μg ml^−1^) compared with labour with opioid and metoclopramide (11.5 [5.7] μg ml^−1^)
*Influence of epidural*							
Carp *et al.,* 1992^70^	Pregnant (T3) (34) Labour (39) Nonpregnant (20)	Cross-sectional study	Pregnant (T3): Standard meal (20) Unspecified solid food (14) Labour: Epidural bupivacaine alone and unspecified solid food (39) Nonpregnant: Standard meal (20)	Gastric US	Liquids and solids	Following an overnight fast, ingestion of a standard meal 800 g and unrestricted clear fluids Gastric US then performed in RLSR at 45 degrees or semirecumbent position every 2 h Following an unspecified fast, ingestion of unspecified solid food and unrestricted clear fluids Gastric US in an unspecified position then performed once at varying time intervals Following a variable fast in labour, patients received epidural bupivacaine 0.25%, 10 ml Gastric US then performed in RLSR at 45 degrees once within 1 h of epidural placement	Solid food detected at 0–24 h in stomach of 71.8% of patients in labour with epidural bupivacaine alone, independent of the time from last oral intake, compared with no solid food present at 4 h in stomach in T3 and nonpregnant
Vallejo *et al.,* 2013^37^	Labour (156)	Randomised controlled trial	Protein (72) Water (84)	Gastric US	Liquids	Following a fast of at least 4 h, patients given epidural bupivacaine 0.08%, 8 ml with fentanyl 100 μg and subsequently received bupivacaine 0.08% with fentanyl 2 μg ml^−1^ as a continuous infusion at 8 ml h^−1^ with patient-controlled epidural boluses of 8 ml Patients ingested protein drink 325 ml (protein 30 g and sugar 1 g with a total of 160 kcal) and water or water alone Gastric US performed in RLSR at 45 degrees before and after ingestion and then every 10 min for 2 h	No difference in gastric half emptying time or incidence of nausea and vomiting between labour with protein and labour with water
Bataille *et al.,* 2014^38^	Pregnant (T3) (6) Labour (60)	Prospective cohort study	–	Gastric US	Liquids	Following an unspecified fast, patients given epidural ropivacaine 0.1%, 10 ml with sufentanil 0.5 μg ml^−1^ and subsequently received ropivacaine 0.1%, with sufentanil 0.5 μg ml^−1^ as a continuous infusion at 5 ml h^−1^ with patient-controlled epidural boluses of 5 ml Gastric US performed in semirecumbent position at 45 degrees at time of epidural request and once cervix fully dilated	In T3, median antral CSA was 90 mm^2^ following an overnight fast and 409 mm^2^ subsequent to ingestion of liquids, justifying use of 320 mm^2^ as the cut-off value for a stomach at risk Gastric antral CSA increased in labour at time of epidural request (319 [77.8] mm^2^) compared with full cervical dilation (203 [34.5] mm^2^) Incidence of gastric antral CSA >320 mm^2^ increased in labour at time of epidural request (50%) compared with full cervical dilatation (13%)
Rousset *et al.,* 2020^39^	Labour (125)	Randomised controlled trial	Fasted (62) Fluids (63)	Gastric US	Liquids	Patients given epidural and patient-controlled epidural boluses of ropivacaine 0.1% and sufentanil 0.5 μg ml^−1^ Patients remained fasted or received apple juice up to 400 ml Gastric US then performed in semirecumbent position at 45 degrees at baseline and full cervical dilatation or 1.5 h Empty stomach if gastric antral CSA <300 mm^2^	No difference in incidence of patients with empty stomach between fasted and fluids in labour
Bouvet *et al.,* 2022^71^	Pregnant (T3) (10) Labour (20) Nonpregnant (10)	Cross-sectional study	No epidural (10) Epidural ropivacaine and opioid (10)	Gastric US	Solids	Following a fast of at least 1 h for clear fluids and 6 h for solids, ingestion of a standard meal (carbohydrate 21.2 g, fat 2.2 g, and protein 3.8 g with a total of 120 kcal) Standard meal ingested 1 h subsequent to initiation of epidural analgesia and patients received epidural ropivacaine 0.1% with sufentanil 0.25 μg ml^−1^ as a continuous infusion at 3 ml h^−1^ with patient-controlled epidural boluses of 5 ml Gastric US then performed in RLSR at 45 degrees at 15 min and 1, 1.5, and 2 h	Gastric half emptying time of labour without epidural not calculated Gastric half emptying time increased in labour with epidural ropivacaine and opioid (72 [56.3] min) compared with T3 (35 [20] min) and nonpregnant (43 [23.7] min) Gastric emptying fraction at 1 h decreased in labour without epidural (4% [3.7%]) or with epidural ropivacaine and opioid (9% [11.1%]) compared with T3 (34% [22.2%]) and nonpregnant (27% [15.6%]) Gastric emptying fraction at 1.5 h reduced in labour without epidural (7% [3.7%]) compared with labour with epidural ropivacaine and opioid (31% [16.3%]), T3 (45% [18.5%]) and nonpregnant (52% [11.1%])
Liu *et al.,* 2 2023^40^	Labour (120)	Prospective cohort study	Epidural ropivacaine and opioid (90) Nonpharmacological methods of analgesia, intravenous midazolam with fentanyl, or both (30)	Gastric US	Liquids and solids	Following the need for analgesia, ingestion of a standard meal (carbohydrate 72.7 g, fat 15.7 g and protein 11.6 g with a total of 70 kcal) and water 300 ml before provision of pain relief Gastric US then performed in RLSR at 45 degrees subsequent to ingestion and before analgesia and at 1 and 2 h	No difference in gastric antral CSA and gastric emptying at 1 h and 2 h between labour with epidural ropivacaine and opioid and labour with nonpharmacological methods of analgesia and/or intravenous midazolam with fentanyl
*Influence of epidural constituents*							
Wright *et al.,* 1992^41^	Labour (30)	Randomised controlled trial	Epidural bupivacaine alone (15) Epidural bupivacaine and opioid (15)	Paracetamol absorption	Drug and liquids	Following a fast of at least 4 h, patients received epidural bupivacaine 0.375%, 10 ml or bupivacaine 0.375%, 10 ml with fentanyl 100 μg and subsequent bupivacaine 0.375%, 8 ml on request 15 min later, ingestion of paracetamol 1.5 g with water 100 ml Blood sampling then performed every 15–30 min for 3 h	Peak plasma paracetamol concentration decreased in labour with epidural bupivacaine and opioid (18 [3.3] μg ml^−1^) compared with labour with epidural bupivacaine alone (27 [11.3] μg ml^−1^) Time to peak plasma paracetamol concentration increased in labour with epidural bupivacaine and opioid (75 [36.6] min) compared with labour with epidural bupivacaine alone (60 [50.1] min) No difference in area under plasma paracetamol curve at 1 h between labour with epidural bupivacaine alone and labour with epidural bupivacaine and opioid
Ewah *et al.,* 1993^43^	Labour (36)	Randomised controlled trial	Epidural lower concentration bupivacaine alone (4) Epidural lower concentration bupivacaine and higher dose fentanyl (4) Epidural lower concentration bupivacaine and higher dose diamorphine (4) Epidural higher concentration bupivacaine alone (8) Epidural higher concentration bupivacaine and lower dose fentanyl (8) Epidural higher concentration bupivacaine and lower dose diamorphine (8)	Paracetamol absorption	Drug and liquids	Following an unspecified fast, patients received epidural bupivacaine 0.25%, 10 ml and subsequent bupivacaine 0.125% or 0.25%, 10 ml with fentanyl 50 or 100 μg or diamorphine 2.5 or 5 mg 30 min later, ingestion of paracetamol 1.5 g Blood sampling then performed every 15 min for 1.5 h	Peak plasma paracetamol concentration decreased in labour with lower concentration bupivacaine and higher dose diamorphine (6.4 [1.0] μg ml^−1^) compared with labour with lower concentration bupivacaine alone (24.4 [4.4] μg ml^−1^) Time to peak plasma paracetamol concentration increased in labour with higher concentration bupivacaine and lower dose fentanyl (75.6 [7.4] min) compared with labour with higher concentration bupivacaine alone (45.6 [5.6] min), increased in labour with lower concentration bupivacaine and higher dose fentanyl (90 [16.6] min) relative to labour with lower concentration bupivacaine alone (45.6 [5.6] min), and increased in labour with lower concentration bupivacaine and higher dose diamorphine (257.5 [20] min) compared with labour with lower concentration bupivacaine alone (45.6 [5.6] min) No difference in peak plasma paracetamol concentration or time to peak plasma paracetamol concentration between all other permutations of concentration of bupivacaine and dose of opioid in labour
Zimmermann *et al.,* 1996^44^	Labour (28)	Randomised controlled trial	Epidural bupivacaine alone (14) Epidural bupivacaine and opioid (14)	Paracetamol absorption	Liquids	Following an unspecified fast, patients given epidural bupivacaine 0.125%, 10 ml with or without fentanyl 50 μg and subsequently received bupivacaine 0.125% with or without fentanyl 2 μg ml^−1^ as a continuous infusion at 10 ml h^−1^ 2 h later, ingestion of paracetamol 20 mg kg^−1^ with water 150 ml Blood sampling then performed every 15 min for 2.5 h	No difference in area under plasma paracetamol curve at 45 min or 1.5 h, peak plasma paracetamol concentration, or time to peak plasma paracetamol concentration between labour with epidural bupivacaine alone and labour with epidural bupivacaine and opioid
Kelly *et al.,* 1997^45^	Labour (101)	Randomised controlled trial	Epidural higher concentration bupivacaine alone (33) Epidural lower concentration bupivacaine and opioid (34) Intrathecal bupivacaine and opioid (34)	Paracetamol absorption	Drug and liquids	Following a fast of at least 3 h, patients received epidural bupivacaine 0.375%, 10 ml, bupivacaine 0.25%, 10 ml with fentanyl 50 μg or intrathecal bupivacaine 2.5 mg with fentanyl 25 μg 15 min later, ingestion of paracetamol 1.5 g with water 100 ml Blood sampling then performed every 15–30 min for 1.5–2.5 h	Area under plasma paracetamol curve at 1.5 h decreased in labour with intrathecal bupivacaine and opioid (430 [491] μg ml^−1^ min^−1^) compared with labour with epidural higher concentration bupivacaine alone (736 [504] μg ml^−1^ min^−1^) and labour with epidural lower concentration bupivacaine and opioid (672 [453] μg ml^−1^ min^−1^) Peak plasma paracetamol concentration reduced in labour with intrathecal bupivacaine and opioid (13.4 [8.8] μg ml^−1^) compared with labour with epidural lower concentration bupivacaine and opioid (17.9 [8.1] μg ml^−1^) Time to peak plasma paracetamol concentration increased in labour with intrathecal bupivacaine and opioid (120 [41.3] min) compared with labour with epidural higher concentration bupivacaine alone (90 [41.3] min) and labour with epidural lower concentration bupivacaine and opioid (82.5 [41.3] min)
Porter *et al.,* 1997^46^	Labour (55)	Randomised controlled trial	Epidural higher concentration and lower volume bupivacaine alone (14) Epidural higher concentration and volume bupivacaine alone (13) Epidural lower concentration and volume bupivacaine and opioid (14) Epidural lower concentration and higher volume bupivacaine and opioid (14)	Paracetamol absorption	Drug and liquids	Following an unspecified fast, patients given epidural bupivacaine 0.25%, 10–15 ml and subsequently received bupivacaine 0.0625% or 0.125% with or without fentanyl 2.5 μg ml^−1^ as a continuous infusion at 10–12 ml h^−1^ Once 30 or 40–50 ml of epidural solution had been administered, ingestion of paracetamol 1.5 g with unspecified volume of water Blood sampling then performed every 15–30 min for 1.5 h	Time to peak plasma paracetamol concentration increased in labour with epidural lower concentration and higher volume bupivacaine and opioid (76.1 [23.9] min) compared with epidural higher concentration and volume bupivacaine alone (54.2 [27.8] min) No difference in area under plasma paracetamol concentration curve, peak plasma paracetamol concentration, and time to peak plasma paracetamol concentration between all other permutations of concentration and volume of bupivacaine with or without opioid
Fiszer *et al.,* 2022^47^	Labour (80)	Randomised controlled trial	Epidural bupivacaine and lower dose opioid (40) Epidural bupivacaine and higher dose opioid (40)	Gastric US	Liquids and solids	No restriction on oral intake Patients given epidural bupivacaine 0.1%, 10 ml with fentanyl 25 or 100 μg and subsequently received bupivacaine 0.083%, with fentanyl 1 or 2 μg ml^−1^ as a continuous infusion at 6 ml h^−1^ with patient-controlled epidural boluses of 5 ml Gastric US of performed in semirecumbent position at 45 degrees at 0 and 2 h	No difference in gastric antral CSA between 0 and 2 h for labour with epidural bupivacaine and lower dose opioid and labour with epidural bupivacaine and higher dose opioid No difference in gastric antral CSA at 2 h between labour with epidural bupivacaine and lower dose opioid and labour with epidural bupivacaine and higher dose opioid, regardless of fasting status
*Carbohydrate drink*							
Ni *et al.,* 2024^48^	Labour (40)	Randomised controlled trial	Carbohydrate drink (20) High energy semifluid solid drink (20)	Gastric US	Liquids and solids	Following a fast of at least 2 h for clear fluids, 6 h for light meal, and 8 h for fatty meal, carbohydrate drink 300 ml or high energy semifluid solid drink 300 ml ingested 10 min subsequent to initiation of epidural analgesia with unspecified constituents Gastric US then performed in RLSR at 45 degrees before ingestion and at 5 and 30 min and 1, 1.5, and 2 h subsequent to ingestion	Gastric antral CSA decreased at 30 min and 1 and 1.5 h with carbohydrate drink compared with high energy semifluid solid drink in labour No difference in gastric antral CSA at 2 h between carbohydrate drink and high energy semifluid solid drink in labour
*Miscellaneous drugs*							
Howard *et al.,* 1973^49^	Labour (25)	Randomised controlled trial	I.V. oxytocin infusion I.M. meperidine if needed for analgesia Placebo (12) Metoclopramide (13)	Nasogastric sampling and dye dilution	Liquids	Following a fast of at least 18 h, gastric contents aspirated before ingestion of water 750 ml containing phenol red 30 μg ml^−1^ Nasogastric sampling then performed every 10 min for 2–3 h	Gastric half emptying time decreased in labour with metoclopramide (51 min and sd unspecified) compared with labour without metoclopramide (141 min and sd unspecified) No difference in gastric volume at 10 min between placebo and metoclopramide Gastric volume at 20 min reduced in labour with metoclopramide (451 [150] ml) compared with labour without metoclopramide (613 [41] ml) Gastric volume at 30 min reduced in labour with metoclopramide (363 [119] ml) compared with labour without metoclopramide (567 [42] ml)
Frame *et al.,* 1984^50^	Labour (30)	Randomised controlled trial	Epidural with unspecified components Placebo (15) Naloxone (15)	Paracetamol absorption	Drug and liquids	Following a fast of at least 4 h, patients given i.m. meperidine 100 mg and then epidural analgesia with unspecified constituents They then received i.v. naloxone 1.2 mg or saline 3 ml followed by ingestion of paracetamol 1.5 g and water 100 ml Blood sampling then performed every 15 min for 1.5 h	Area under plasma paracetamol curve at 30 min decreased in labour with naloxone (2.1 [0.5] μg ml^−1^ h^−1^) compared with labour without naloxone (1.5 [0.3] μg ml^−1^ h^−1^) No difference in area under plasma paracetamol curve at 1.5 h or peak plasma paracetamol concentration between naloxone and placebo
**Postpartum and nonpregnant after postpartum**							
*Changes over time in postpartum*							
Vial *et al.,* 2017^59^	Labour (100) Postpartum (100)	Prospective cohort study	Postpartum: <45 min following neonatal delivery	Gastric US	Liquids	Following an unspecified fast, patients given epidural levobupivacaine 0.125%, 10 ml with sufentanil 10 μg and subsequently received levobupivacaine 0.125% with sufentanil 0.5 μg ml^−1^ as a continuous infusion at 5 ml h^−1^ with patient-controlled epidural boluses of 5 ml Gastric US performed in semirecumbent position at 45 degrees then performed immediately following epidural insertion and within 45 min following neonatal delivery Empty stomach if gastric antral CSA <381 mm^2^	30% of patients had clear fluids and none had solids following epidural insertion Clear fluid ingestion in labour was not a risk factor for the presence of full stomach following neonatal delivery 12.2% of patients with empty stomach at epidural insertion had full stomach subsequent to neonatal delivery and 24.4% of patients with full stomach at epidural insertion had empty stomach following neonatal delivery
Gin *et al.,* 1991^60^	Postpartum (22)	Prospective cohort study	1 day, 3 days, and 6 weeks following neonatal delivery	Paracetamol absorption	Drug and liquids	Following an overnight fast, ingestion of paracetamol 1.5 g with water 100 ml Blood sampling then performed every 15–30 min for 2 h	Area under plasma paracetamol curve at 1 h increased at 6 weeks compared with 1 day and 3 days postpartum (numbers unspecified) Area under plasma paracetamol curve at 2 h increased at 6 weeks (51.1 [10.4] μg ml^−1^ h^−1^) compared with 1 day (43.8 [5.8] μg ml^−1^ h^−1^) and 3 days postpartum (42.3 [5] μg ml^−1^ h^−1^) No difference in peak plasma paracetamol concentration between 1 day, 3 days, and 6 weeks postpartum
*Regional anaesthesia or analgesia with no opioid vs opioid in postpartum*							
King *et al.,* 2004^61^	Postpartum (40)	Randomised controlled trial	Intrathecal bupivacaine alone (20) Intrathecal bupivacaine and opioid (20)	Paracetamol absorption	Drug and liquids	Following an overnight fast, spinal anaesthesia with intrathecal bupivacaine 0.5%, 2.75 ml with or without diamorphine 300 μg for Caesarean delivery 30 min postoperatively, patients received paracetamol 1.5 g with water 100 ml Blood sampling then performed every 15–30 min for 2 h	No difference in area under plasma paracetamol curve at 2 h or peak plasma paracetamol concentration between intrathecal bupivacaine and opioid and intrathecal bupivacaine alone Time to peak plasma paracetamol concentration increased with intrathecal bupivacaine and opioid (72.6 [41.9] min) compared with intrathecal bupivacaine alone (41.8 [20.8] min)
Geddes *et al.* 1991^62^	Postpartum (30)	Randomised controlled trial	Epidural bupivacaine alone (15) Epidural bupivacaine and opioid (15)	Paracetamol absorption	Drug and liquids	Following an unspecified fast, ingestion of ranitidine 150 mg Epidural anaesthesia with bupivacaine 0.5% for Caesarean delivery Subsequent to Caesarean delivery, patients received epidural bupivacaine 0.25%, 8 ml with or without fentanyl 100 μg and ingested paracetamol 1.5 g with water 50 ml Blood sampling then performed every 15 min for 1.5 h	Area under plasma paracetamol curve at 45 min decreased with epidural bupivacaine and opioid (2.5 [3.9] mg ml^−1^ h^−1^) compared with epidural bupivacaine alone (7.5 [3.2] mg ml^−1^ h^−1^) No difference in area under plasma paracetamol curve at 1.5 h or peak plasma paracetamol concentration between epidural bupivacaine and opioid and epidural bupivacaine alone
*Postpartum vs other groups*							
Wald *et al.,* 1982^63^	Pregnant (T3) (15) Postpartum (15)	Prospective cohort study	Postpartum: 4–6 weeks following neonatal delivery	Breath hydrogen analysis	Drug and liquids	Following an overnight fast, ingestion of lactulose 11.5 g with water 100 ml Breath sampling then performed every 10 min	OCTT increased in T3 (131 [54] min) compared with postpartum (93 [27] min)
Lawson *et al.,* 1985^72^	Pregnant (T1–3) (42) Postpartum (17)	Prospective cohort study	Pregnant: T1(8) T2 (12) T3 (22) Postpartum: 4–8 weeks following neonatal delivery	Breath hydrogen analysis	Drug and liquids	Following an overnight fast, ingestion of lactulose 10 g with water 100 ml Breath sampling then performed every 15 min for 4 h	OCTT increased in T2 (125 [48] min) and T3 (137 [58] min) compared with postpartum (75 [33] min)
O'Sullivan *et al.,* 1987^73^	Pregnant (T3) (18) Postpartum (15) Nonpregnant (13)	Cross-sectional study	Postpartum: <60 min following neonatal delivery No opioid in labour (10) Opioid in labour (5)	Epigastric impedance	Liquids	Following a fast of at least 4 h, ingestion of water or diluted orange concentrate 500 ml Electrical impedance then measured through electrodes on the epigastrium	Gastric half emptying time increased in postpartum (13.0 [7.4] min) compared with T3 (7.2 [2.5] min) and nonpregnant (8.3 [3.2] min) Gastric half emptying time increased in postpartum following opioid in labour (18.2 [8.9] min) compared with postpartum without opioid in labour (10.3 [4.4] min)
Sandhar *et al.,* 1992^65^	Pregnant (T3) (10) Postpartum (20)	Prospective cohort study	Postpartum: 2–3 days (10) 6 weeks (10)	Epigastric impedance	Drug and liquids	Following a 4-h fast and ranitidine 150 mg, ingestion of water 400 ml Electrical impedance then measured through electrodes on the epigastrium	No difference in gastric half emptying time between T3 and postpartum
Whitehead *et al.,* 1993^21^	Pregnant (T1–3) (64) Postpartum (55) Nonpregnant (32)	Prospective cohort study	Pregnant: T1 (18) T2 (10) T3 (36) Postpartum: 2 h–5 days following neonatal delivery In those 2 h postpartum (17), 4 had i.m. meperidine and 8 received epidural analgesia	Paracetamol absorption	Drug and liquids	Following a fast of least 4 h, ingestion of paracetamol 1.5 g with water 50 ml Blood sampling then performed every 10–30 min for 2 h	Area under plasma paracetamol concentration curve decreased in postpartum at 2 h (3.8 [4.1] mg L^−1^ h^−1^) compared with nonpregnant (13.5 [5.8] mg L^−1^ h^−1^) Peak plasma paracetamol concentration reduced in postpartum at 2 h (12.5 [5.1] mg L^−1^) compared with nonpregnant (20.8 [14] mg L^−1^) Time to peak plasma paracetamol concentration increased in postpartum at 2 h (120 [22.5] min) compared with nonpregnant (40 [27.5] min) No difference in area under plasma paracetamol curve at 120 min, peak plasma paracetamol concentration and time to peak plasma paracetamol concentration between postpartum at 1–5 days and nonpregnant
Nimmo *et al.,* 1975^35^	Labour (46) Postpartum (10)	Cross-sectional study	Labour: No opioid (12) Opioid (34) Postpartum: 2–5 days following neonatal delivery	Paracetamol absorption	Drug and liquids	Following a fast of at least 4 h and a variable period following opioid, ingestion of paracetamol 1.5 g with water 200 ml Blood sampling then performed every 15 min for 1.5 h and subsequently every 1–2 h up to 8 h	No difference in peak plasma paracetamol concentration between no opioid in labour and postpartum
Miller *et al.,* 1986^66^	Postpartum (12) Nonpregnant (14)	Prospective cohort study	Postpartum: 36–110 h following neonatal delivery	Paracetamol absorption	Drug and liquids	Following a fast of at least 4 h, ingestion of paracetamol 1.5 g with unspecified amount of water Blood sampling then performed every 15–30 min for 2 h	Area under plasma paracetamol curve decreased in postpartum compared with nonpregnant (numbers unspecified) Peak plasma paracetamol concentration decreased in postpartum (18.7 [8.1] μg ml^−1^) compared with nonpregnant (24.6 [10.4] μg ml^−1^)
Lam *et al.,* 1993^67^	Postpartum (100) Nonpregnant (50)	Randomised controlled trial	Postpartum: <5 days following neonatal delivery Fasted (50) Nonfasted (50)	Nasogastric sampling	Liquids	Postpartum patients having tubal ligation and nonpregnant patients scheduled for laparoscopic surgery Following an overnight fast, ingestion of water 150 ml in half the postpartum patients Standardised general anaesthetic Nasogastric sampling then performed	No difference in gastric volume between fasted postpartum, nonfasted postpartum, and nonpregnant
Jayaram *et al.,* 1997^68^	Postpartum (48) Nonpregnant (45)	Cross-sectional study	Postpartum: <1 day postpartum following neonatal delivery	Gastric US	Solids	First study: Postpartum patients having tubal ligation and nonpregnant patients scheduled for gynaecological surgery Following a fast of at least 6 h, gastric US then performed in erect position before induction of anaesthesia Second study: Postpartum patients having recently delivered and nonpregnant patients not scheduled for surgery Following an unspecified fast, ingestion of a standard meal (carbohydrate 115–120 g, fat 25–30 g, and protein 15–20 g) Gastric US then performed in semirecumbent position at 0 and 4 h	First study: Solid food detected in stomach of 39.3% of postpartum patients compared with no solid food present in stomach in nonpregnant at 6 h Second study: Solid food detected in stomach of 95% of postpartum patients compared with solid food present in stomach of 19% of nonpregnant patients at 4 h
Scrutton *et al.,* 1997^69^	Postpartum (6) Nonpregnant (10)	Prospective cohort study	Postpartum: 18–24 h following neonatal delivery	Gastric US and scintigraphy	Drug, liquids and solids	Following an overnight fast, ingestion of carbohydrate 200 ml labelled with 15 Mbq ^99^Tc^m^ Dowex resin and water 200 ml Gastric US of then performed in supine position every 15–30 min for 2 h and scintigraphy imaging every 1–30 min for 2 h	No difference in rate of gastric emptying between postpartum and nonpregnant
*Nonpregnant after postpartum vs other groups*							
Stanley *et al.,* 1995^30^	Pregnant (T1–3) (29) Nonpregnant after postpartum (10)	Prospective cohort study	Pregnant: T1 (10) T2 (9) T3 (10) Nonpregnant after postpartum: 8 weeks following neonatal delivery (10)	Paracetamol absorption	Liquids and solids	Following an 8-h fast, ingestion of a standard meal (carbohydrate 59 g, fat 21.2 g, and protein 17.9 g) with water 200 ml and paracetamol 1.5 g with water 50 ml Blood sampling then performed every 20 min for 2.7 h	No difference in peak plasma paracetamol concentration or time to peak plasma paracetamol concentration between T1, T2, T3, and nonpregnant after postpartum
Szilagyi *et al.,* 1996^28^	Pregnant (T2–3) (30) Nonpregnant after postpartum (27) Nonpregnant (17)	Prospective cohort study	Pregnant: Unspecified mix of T2–3 Nonpregnant after postpartum: 3–14 months following neonatal delivery	Breath hydrogen analysis	Drug and liquids	Following an overnight fast, ingestion of lactulose 10 g with water 100 ml Breath sampling then performed every 10 min for 3 h	OCTT increased in pregnant (99.2 [7.8] min) and nonpregnant after postpartum (68.5 [6.4] min) compared with nonpregnant (63.5 [8.7] min)
Chiloiro *et al.,* 2001^32^	Pregnant (T1 and 3) (22) Nonpregnant after postpartum (11)	Prospective cohort study	Pregnant: T1 (11) T3(11) Nonpregnant after postpartum: 4–6 months following neonatal delivery	Breath hydrogen analysis and gastric US	Drug and liquids	Following an overnight fast, ingestion of lactulose 18 g with water 180 ml Breath sampling then performed every 10 min for 4–6 h and gastric US in erect position every 10 min until stomach empty	OCTT increased in T3 (100 [95% IQR 50.5–240] min) compared with nonpregnant after postpartum (70 [95% IQR 40.5–240] min) No difference in gastric emptying between T1, T3 and nonpregnant after postpartum

These different methods of examining gastric emptying are now summarised in brief. Gastric ultrasound involves the repeated acquisition of sonographic images to visualise the gastric antrum, the more dependent area of the stomach, and leads to qualitative and quantitative assessment.^15^^,^^16^ Qualitative evaluation uses the Perlas grading scale. For patients who are nonpregnant, in T1, or postpartum, gastric ultrasound is carried out in the supine and right lateral position and for who those in T2 and T3, it is conducted in the right lateral semirecumbent and semirecumbent position. Gastric contents shift under the influence of gravity, and ultrasound should be performed in precisely defined positions to allow reliable imaging and consistent measurement.^74^ The right lateral semirecumbent and semirecumbent position are preferred in pregnancy to avoid the effect of supine aortocaval compression^75^ and displace the gravid uterus away from the ultrasound path.^70^ In Grade 0 an empty gastric antrum is appreciated in the right lateral semirecumbent and semirecumbent position, in Grade 1 an empty gastric antrum is seen in the semirecumbent position although clear fluids are visible in the right lateral semirecumbent position, in Grade 2 clear fluids are viewed in both of these positions, and in Grade 3 thick fluids or solids are present in the stomach. Quantitative evaluation utilises the cross-sectional area of the gastric antrum to estimate the volume in the stomach.^76^ If administered orally, paracetamol is absorbed poorly in the stomach but well in the duodenum and proximal jejunum. Given that gastric emptying is hence the rate-limiting step in the delivery of the drug to its absorption site, the speed of paracetamol absorption into the blood indirectly reflects the rate of gastric emptying. The area under plasma paracetamol curve and peak plasma paracetamol concentration are proportional and the time to peak paracetamol concentration is inversely proportional to the rate of gastric emptying. Nasogastric sampling directly measures the volume of the stomach. In the dye dilution method, dye is progressively added to the stomach and the volume is then calculated from the resulting change in its concentration.^64^ With breath hydrogen analysis, the oral administration of lactulose leads to its unmetabolised transit through the stomach and intestine, as no naturally occurring lactulose enzyme is present in these regions, and the subsequent metabolism of lactulose to hydrogen by bacteria in the caecum.^77^ The resulting increase in the expired hydrogen concentration is therefore related to the orocaecal transit time rather than only the rate of gastric emptying. In epigastric impedance, the change in resistance to an alternating current is measured over time.^78^ The use of barium X-ray enables the direct visualisation of barium as it empties from the stomach and transits through the gastrointestinal tract. In breath ^13^C-octanoic acid analysis, the medium-chain fatty acid once ingested is emptied from the stomach and absorbed in the proximal small intestine.^16^ It is then transported to the liver and metabolised to carbon dioxide. The carbon dioxide is labelled with ^13^C as a specific marker of octanoic acid oxidation and subsequently excreted from the lungs, where it is measured. With electrogastrography, the myoelectrical signals from the stomach are recorded from the skin surface and facilitate the monitoring of gastric activity and motility.^78^ The radiotelemetry pH pill signals its emptying from the stomach with the sudden change of pH from acidic in the gastric milieu to almost alkaline in the duodenum.^16^ Scintigraphy has been recognised as the gold standard to directly quantify gastric emptying and involves the utilisation of a gamma camera to identify the radiation emitted from the ingested standard meal.

### Pregnancy

#### Gastric emptying in the first trimester

Compared with patients who were nonpregnant, women in T1 were demonstrated in four studies and two studies at moderate risk of bias to have either a decrease with water^22^^,^^24^^,^^26^^,^^27^ or no difference with water or solids^21^^,^^29^ in gastric emptying, respectively. One of these studies was a randomised controlled trial and found gastric emptying with water to reduce in patients in T1 relative to women who were nonpregnant.^22^ The method to investigate gastric emptying was paracetamol absorption with water^21^^,^^22^^,^^24^^,^^26^^,^^27^ and breath ^13^C-octanoic acid analysis with solids.^29^ One observational study, that was at moderate risk of bias, noted no difference in gastric emptying with water between T1, T2, and T3^26^ and another observational study, which was at high risk of bias, revealed the orocaecal transit time with water to increase with the progression of pregnancy.^72^

#### Gastric emptying delayed in the second trimester

Compared with patients who were nonpregnant, women in T2 were found by paracetamol absorption in two observational studies at moderate risk of bias to have no difference in gastric emptying with water or water and solids.^21^^,^^26^

#### Gastric emptying delayed in the third trimester

Compared with patients who were nonpregnant, women in T3 were demonstrated in seven studies, including one nonrandomised controlled trial^23^ and one at low risk of bias,^71^ to have no difference in gastric emptying with water, diluted orange concentrate, liquid formula, or solids.^21^^,^^23^^,^^25^^,^^26^^,^^64^^,^^71^^,^^73^ One observational study, however, found gastric emptying to decrease with water and solids in patients in T3 relative to women who were nonpregnant.^5^ The method to investigate gastric emptying was paracetamol absorption in two studies^21^^,^^26^ and varied in the remaining ones.^5^^,^^23^^,^^25^^,^^64^^,^^71^^,^^73^

In women who were T3, the ingestion of liquid formula following a fast of 2 h for clear liquids and 6 h for solids led to all patients having a gastric volume of <80 ml on gastric ultrasound at 2 h.^42^ Further, carbohydrate drink^51^^,^^52^ and tea with milk^53^ resulted in no difference in gastric antral cross-sectional area at this same time point compared with baseline or water. Subsequent to the ingestion of solids, no food was present in the stomach at 4 h.^70^

#### Effect of medications on gastric emptying in pregnancy

In patients who were T1, glycopyrronium decreased the area under plasma paracetamol curve with water compared with no premedication and atropine.^22^ In women who were T3, no difference in gastric half emptying time with water was found between the antacids, magnesium trisilicate, and sodium citrate.^23^

### Caesarean delivery

In patients who were fasted, granisetron decreased the gastric antral cross-sectional area at 1 h compared with baseline.^55^ Metoclopramide reduced the gastric antral cross-sectional area at 1 h relative to baseline^56^ and resulted in an increase in the plasma paracetamol concentration at this same time point.^36^ Following Caesarean delivery, two randomised controlled trials at moderate risk of bias investigated by paracetamol absorption the effect of the constituents of the neuraxial technique on gastric emptying with water in the immediate postpartum period.^61^^,^^62^ Compared with intrathecal bupivacaine alone, intrathecal diamorphine in addition to bupivacaine increased the time to peak plasma paracetamol concentration, although no difference was found in regard to the area under plasma paracetamol curve at 2 h and the peak plasma paracetamol concentration.^61^ Relative to epidural bupivacaine alone, epidural fentanyl with bupivacaine reduced the area under plasma paracetamol curve at 45 min, but no difference was shown with respect to the area under plasma paracetamol curve at 1.5 h and the peak plasma paracetamol concentration.^62^ Subsequent to Caesarean delivery, the ingestion of carbohydrate drink in a randomised controlled trial at moderate risk of bias increased the gastric antral cross-sectional area at 1 h although not 1.5 h relative to water.^52^

### Labour

#### Gastric emptying in labour without an epidural

Compared with patients who were T3 and nonpregnant, women in labour were demonstrated in two observational studies, one at low risk of bias and by gastric ultrasound^71^ and one at high risk of bias and by nasogastric sampling and dye dilution,^64^ to have increased gastric volume with water at 30 min^64^ and decreased gastric emptying fraction with solids at 1 h.^71^ Patients in labour were found by paracetamol absorption in one randomised controlled trial at moderate risk of bias to have a reduced plasma paracetamol concentration with water at 1 h relative to women who were having Caesarean delivery.^36^ In a randomised controlled trial at moderate risk of bias, gastric emptying was noted by gastric ultrasound to decrease with orange juice or coffee and milk compared with maltodextrin and no difference in gastric emptying was revealed between orange juice and coffee with milk.^34^ Intramuscular diamorphine and meperidine were shown by paracetamol absorption in one observational study at moderate risk of bias to reduce the peak plasma paracetamol concentration and increase the time to peak plasma paracetamol with water relative to no opioid in labour.^35^ These findings for intramuscular meperidine were supported by the results of a randomised controlled trial.^36^ The inhibitory effect of intramuscular diamorphine and meperidine on gastric emptying was not reversed by metoclopramide in one observational study.^35^ This effect with meperidine, however, was reversed in a larger randomised controlled trial.^36^

#### Gastric emptying in labour with an epidural

Compared with patients who were T3 and nonpregnant, epidural bupivacaine alone for women in labour was demonstrated by gastric ultrasound in one observational study at high risk of bias to increase the risk of solid food detection in the stomach, independent of the time from last oral intake, from 0% to 71.8% at 0–24 h.^70^ Relative to patients who were T3 or nonpregnant, epidural ropivacaine and opioid for women in labour was found by gastric ultrasound in one observational study at low risk of bias to lead to an increased gastric emptying time with solids.^71^ Importantly, in the same observational study, the gastric emptying fraction was increased in labour with epidural ropivacaine and opioid compared with without epidural. In two randomised controlled trials, one at low risk of bias^39^ and one at high risk of bias, the incidence of empty stomach did not differ by gastric ultrasound between fasted and apple juice in patients who had epidural ropivacaine and opioid,^39^ and no difference in gastric emptying was shown between water and water with protein drink in women who received epidural bupivacaine and opioid.^37^ In the presence of an epidural with unspecified constituents, the gastric antral cross-sectional area was decreased at 1.5 h but not 2 h following the ingestion of a carbohydrate drink relative to a high-energy semifluid solid drink in a randomised controlled trial at moderate risk of bias.^48^

The constituents of the epidural solution may have an effect on gastric emptying. In regard to the concentration and volume of local anaesthetic, two randomised controlled trials at moderate risk of bias used the method of paracetamol absorption with water to investigate this.^43^^,^^46^ No difference was demonstrated between epidural lower concentration bupivacaine and epidural higher concentration bupivacaine alone of same volume in the area under plasma paracetamol concentration and the time to peak plasma concentration.^43^ Moreover, no difference was shown between epidural higher concentration and lower volume bupivacaine and epidural higher concentration and volume bupivacaine alone in the area under plasma paracetamol concentration, peak plasma paracetamol concentration, and the time to peak plasma paracetamol concentration.^46^ In relation to the presence of opioid, three randomised controlled trials at moderate risk of bias used the technique of paracetamol absorption with water to investigate this.^41^^,^^43^^,^^44^ On the one hand, in one randomised controlled trial, the administration of an epidural bolus dose of fentanyl 100 μg in addition to bupivacaine decreased the peak plasma paracetamol concentration and increased the time to peak plasma paracetamol concentration compared with bupivacaine alone.^41^ In another randomised controlled trial, the use of an epidural bolus dose of fentanyl 50 μg or 100 μg with bupivacaine increased the time to peak plasma paracetamol concentration and diamorphine 5 mg with bupivacaine reduced the peak plasma paracetamol concentration and increased the time to peak plasma paracetamol concentration.^43^ On the other hand, in one randomised controlled trial, the administration of an epidural bolus dose of fentanyl 50 μg followed by a continuous infusion with fentanyl 2 μg ml^−1^ in addition to bupivacaine did not affect the area under plasma paracetamol curve at 45 min or 1.5 h, peak plasma paracetamol concentration, and the time to peak plasma paracetamol concentration relative to bupivacaine alone.^44^ In the previously mentioned randomised controlled trial, the use of an epidural bolus dose of diamorphine 2.5 mg with bupivacaine did not influence the peak plasma paracetamol concentration and the time to peak plasma paracetamol concentration.^43^ With respect to the dose of opioid, two randomised controlled trials at moderate risk of bias investigated this.^43^^,^^47^ In one randomised controlled trial, epidural fentanyl at a bolus dose of 25 μg followed by a continuous infusion of 1 μg ml^−1^ was compared with a bolus dose of 50 μg and a subsequent continuous infusion of 2 μg ml^−1^.^47^ Indices of gastric emptying with unrestricted liquids and solids did not vary using the method of gastric ultrasound. In another randomised controlled trial, epidural diamorphine at a bolus dose of 2.5 mg did not affect the peak plasma paracetamol concentration and the time to peak plasma concentration, but a bolus dose of 5 mg did reduce the peak plasma paracetamol concentration and increase the time to peak paracetamol concentration.^43^ The use of intrathecal bupivacaine and fentanyl in labour in a randomised controlled trial at moderate risk of bias slowed gastric emptying with water relative to epidural bupivacaine alone and epidural bupivacaine with opioid using the technique of paracetamol absorption.^45^

#### Effect of medications on gastric emptying in labour

In the absence or presence of intramuscular meperidine, metoclopramide decreased the gastric half emptying time and reduced the gastric volume with water at 30 min^49^ and increased the plasma paracetamol concentration at 1 h.^36^ In the presence of an epidural with unspecified constituents, naloxone decreased the area under plasma paracetamol curve with water at 30 min but did not result in any difference in the peak plasma paracetamol concentration.^50^

### Postpartum

Indices related to gastric emptying changed over time in the postpartum period. In one observational study at moderate risk of bias, the area under plasma paracetamol curve increased with water at 2 h at 6 weeks compared with 1 and 3 days postpartum, although no difference was demonstrated between these three time points in regard to the peak plasma paracetamol concentration.^60^ Compared with patients who were T2, women were found by breath hydrogen analysis in one observational study at high risk of bias to have a decreased orocaecal transit time with water at 4–8 weeks postpartum.^72^ Relative to patients who were T3, women were noted by breath hydrogen analysis in two observational studies, one at moderate risk of bias^72^ and one at high risk of bias,^63^ to have a reduced orocaecal transit time with water at 4–8 weeks postpartum.^63^^,^^72^ In another observational study at moderate risk of bias, however, no difference in epigastric impedance with water was revealed between patients who were T3 and women 2 days to 6 weeks postpartum.^65^ No difference was shown in one observational study at moderate risk of bias between patients in labour without opioid and women who were 2–5 days postpartum with respect to the peak plasma paracetamol concentration with water.^35^ On the one hand, compared with patients who were nonpregnant, women were found by paracetamol absorption in one observational study at moderate risk of bias to have a decreased area under plasma paracetamol curve and peak plasma paracetamol concentration with water at 36–110 h postpartum.^66^ In an observational study at high risk of bias, subsequent to the ingestion of solids, patients who were nonpregnant did not have solids present in the stomach relative to 39.3% of women at <1 day postpartum who did at 6 h.^68^ On the other hand, compared with patients who were nonpregnant, women were shown by two observational studies, one at moderate risk of bias and by nasogastric sampling with or without water^67^ and one at moderate risk of bias and by gastric ultrasound and scintigraphy with carbohydrate drink,^69^ to have no difference in the gastric volume or the rate of gastric emptying at <5 days postpartum.^67^^,^^69^

### Nonpregnant after postpartum

Compared with patients in T1, T2, and T3, women were found by paracetamol absorption in one observational study at moderate risk of bias to have no difference in the peak plasma paracetamol concentration or the time to peak plasma paracetamol concentration with water and solids at 8 weeks postpartum.^30^ Relative to patients in T1 and T3, women were shown by gastric ultrasound in one observational study at moderate risk of bias to have no difference in gastric emptying with water at 4–6 months postpartum.^32^ The orocaecal transit time with water, however, was increased in patients who were T3 compared with women at 4–6 months postpartum^32^ and in patients who were 3–14 months postpartum relative to nonpregnant women.^28^

## Discussion

In this narrative review, compared with patients who were not pregnant, gastric emptying in T1 was decreased with water in the majority of studies and not different with solids in one study. Gastric emptying in T2 and T3, relative to the nonpregnant phase, was not demonstrated to be different with water or solids in two studies and most studies, respectively. In the context of T3 and Caesarean delivery, the ingestion of carbohydrate drink or tea with milk *vs* continued fasting or water led to no difference in the gastric cross-sectional area at 2 h and, following the ingestion of solids, no food was present in the stomach at 4 h. Gastric emptying was delayed with neuraxial local anaesthetic and opioid compared with local anaesthetic alone. In the setting of labour without an epidural, gastric emptying was reduced with water or solids compared with women who were not pregnant or T3 and was slowed with orange juice or coffee and milk relative to carbohydrate drink. The administration of intramuscular diamorphine or meperidine further decreased gastric emptying. In labour with an epidural using local anaesthetic and opioid, gastric emptying was not different with water *vs* water with protein drink and was reduced with solids compared with the nonpregnant phase or T3, although it was increased with solids relative to labour without an epidural. Gastric emptying was not affected by the concentration of local anaesthetic in the epidural solution. The evidence in regard to the influence of opioid in the epidural solution on gastric emptying was conflicting, either decreasing it or making no difference. In labour, intrathecal local anaesthetic and opioid delayed gastric emptying with water compared with epidural local anaesthetic and opioid. The evidence with respect to the postpartum period was inconsistent. Relative to patients who were not pregnant, one study indicated that gastric emptying was still reduced with water and two studies suggested that it was not different in the first 5 days postpartum. In what follows, we will draw upon the findings of important and landmark studies in gastric ultrasound and interpret the evidence on gastric emptying to inform clinical practice (Table 2).

**Table 2:** Summary of main findings. Inconsistencies in evidence may reflect the unpredictability of gastric emptying in pregnancy. CSA, cross sectional area; T1, first trimester; T2, second trimester; T3, third trimester.

Phase of pregnancy	Method of determination	Principal findings
T1	Gastric emptying	Delay in gastric emptying with water in majority of studies compared with patients who were not pregnant No difference in gastric emptying with solids in one study relative to women who were not pregnant
T2	Gastric emptying	No difference in gastric emptying with water or solids in two studies compared with patients who were not pregnant
T3	Gastric emptying	No difference in gastric emptying with water or solids in most studies compared with patients who were not pregnant Following the ingestion of food, no solids present in stomach at 4 h
T1–3	Gastric ultrasound but no measurement of gastric emptying	Higher gestational age increased likelihood of high-risk gastric contents on ultrasound
Caesarean delivery	Gastric emptying	Preoperative ingestion of carbohydrate drink or tea with milk led to no difference in gastric CSA at 2 h compared with continued fasting or ingestion of water Following the preoperative ingestion of food, no solids present in stomach at 4 h Delay in gastric emptying with intraoperative neuraxial opioid and local anaesthetic relative to local anaesthetic alone
	Gastric ultrasound without measurement of gastric emptying	Studies indicated, in the opinion of the authors, that patients who have followed standard fasting guidelines for elective Caesarean delivery may still have high-risk contents present in stomach
Labour in general	Gastric ultrasound but no measurement of gastric emptying	In the first hour following admission to maternity unit in spontaneous labour or for induction of labour, >66% of patients had high-risk gastric contents Duration of fasting for solids and maximum pain score in last hour of labour associated with presence of high-risk gastric contents Duration of fasting for clear fluids not related to presence of high-risk gastric contents Unrestricted oral intake resulted in 80% of women having high-risk gastric contents Studies suggested, in the opinion of the authors, that women in labour who have eaten solids in the last 8 h still have high-risk contents present in the stomach, and those who have a fasting duration >8 h for solids should be considered for risk stratification by gastric ultrasound before urgent general anaesthesia
Labour in absence of epidural	Gastric emptying	Delay in gastric emptying with water or solids compared with patients who were not pregnant or T3 Delay in gastric emptying with orange juice or coffee and milk relative to carbohydrate drink Delay in gastric emptying with intramuscular diamorphine or meperidine
Labour in presence of epidural	Gastric emptying	Delay in gastric emptying with solids compared with patients who were not pregnant or T3 Increase in gastric emptying with solids relative to women who were in labour without epidural No difference in gastric emptying with water compared with water with protein drink Concentration of local anaesthetic in epidural solution did not affect gastric emptying Conflicting evidence in relation to influence of opioid in epidural solution on gastric emptying Delay in gastric emptying with intrathecal local anaesthetic and opioid relative to epidural local anaesthetic and opioid
Postpartum	Gastric emptying	In first 5 days postpartum, gastric emptying with water was delayed in one study and not different in two studies No difference in gastric emptying after 6 months compared with patients who were pregnant

Our findings indicate that, compared with patients who were not pregnant, gastric emptying was decreased in T1 and not T2 and T3. In agreement with this, the baseline gastric volume on ultrasound was not demonstrated to be different in patients who were in T3 relative to women who were not pregnant in a prospective observational study.^79^ Five of the six studies that assessed gastric emptying in T1, two studies in T2, and two of the eight studies that evaluated gastric emptying in T3 used the paracetamol absorption method.^21^^,^^22^^,^^24^^,^^26^^,^^27^ which has been validated against the gold standard of scintigraphy.^80^ but there are possible drawbacks to this technique. The plasma concentration of paracetamol, once absorbed, is dependent on the volume of distribution and the clearance, and these indices have been found to increase as pregnancy advances.^81^ Given this, the pharmacokinetics of paracetamol in women who were pregnant may have led to a reduction in the area under plasma paracetamol curve and peak plasma paracetamol concentration, predisposing towards a suggestion of delayed gastric emptying with the progression of pregnancy. Importantly, however, the results of the two studies that examined gastric emptying in T3 by paracetamol absorption^21^^,^^26^ were consistent with most of those that used other methods such as epigastric impedance, gastric ultrasound, nasogastric sampling, and radiotelemetry pH pill.^23^^,^^25^^,^^64^^,^^71^^,^^73^ In contrast to these results, increased gestational age was revealed in a prospective observational study to increase the likelihood of high-risk gastric contents on ultrasound, that is fluid >1.5 ml kg^−1^ or the presence of solid food, potentially due to the compression of the stomach by the gravid uterus.^82^

In the context of Caesarean delivery, all of the studies investigated gastric emptying in the elective setting. These patients can hence be considered, in the opinion of the authors, to be in T3 if in the preoperative phase as they are not in labour. Of particular concern, in one prospective observational study, 51 patients in T3 were fasted after a standardised light meal for 6 h and, despite none having solid food detectable in the gastric antrum on ultrasound, 38% of women still had >1.5 ml kg^−1^ of fluid, the threshold below which the risk of pulmonary aspiration is minimal.^83^ Further, in another prospective observational study of patients admitted to the maternity unit, elective Caesarean delivery was found to decrease the likelihood of high-risk gastric contents on ultrasound, that is fluid >1.5 ml kg^−1^ or the presence of solid food, but, despite a median (interquartile range, IQR) fast of 7.5 (3–12) h for clear fluids and 12.75 (11.5–14.5) h for solids, 40% of women still had high-risk gastric contents.^82^ In contrast, in two prospective observational studies it was revealed that, following a fast of 2 h for clear fluids and 6–8 h for solids in T3 before elective Caesarean delivery, only 3.5% of 85 women^84^ and 5% of 103 women^85^ had high-risk gastric contents on ultrasound, that is fluid >1.5 ml kg^−1^. The findings of these studies indicate, in the opinion of the authors, that patients who have followed standard fasting guidelines for elective Caesarean delivery may still have high-risk contents present in the stomach, and gastric ultrasound should be considered in those patients in whom it has been scheduled under general anaesthesia. The risk stratification of women with gastric ultrasound may influence decision-making in the circumstances of category four Caesarean delivery, especially in relation to the choice of anaesthetic technique, and the decision to wake or proceed should failed tracheal intubation occur^86^ Importantly, no evidence, to the knowledge of the authors, supports the notion that prolongation of the preoperative fast in such cases necessarily results in significant emptying of these high-risk gastric contents. Our results provide encouragement that the ingestion of carbohydrate drink or tea with milk leads to no difference in gastric cross-sectional area at 2 h compared with continued fasting or water. Specifically, after a fast of 2 h for clear fluids, 6 h for light meal and 8 h for fatty meal, the median (IQR) gastric volume was 0.8 (0.5–1.0) ml kg^−1^ at baseline and 0.8 (0.6–1.1) ml kg^−1^ at 2 h with a range to 1.4 and 1.6 ml kg^−1^, respectively, following a carbohydrate drink,^51^ and was 0.6–0.7 (0.5–0.9) ml kg^−1^ at baseline and 0.7 (0.5–0.9) ml kg^−1^ and 0.6 (0.5–1.1) ml kg^−1^ at 2 h subsequent to water or tea with milk.^53^ The preoperative ingestion of carbohydrate drink has been advocated by SOAP as one of the elements of enhanced recovery after Caesarean delivery to decrease maternal hypoglycaemia and metabolic stress.^87^ In a randomised controlled trial, the ingestion of carbohydrate drink before Caesarean delivery reduced the required number of boluses and total dose of vasopressor subsequent to combined spinal epidural anaesthesia.^88^ Interestingly, in a prospective observational study, 58 women who were able to drink sips of water once they had been admitted to hospital and before being called for Caesarean delivery were shown to have a noninferior gastric cross-sectional area at the end of the sip-till-send period relative to when they were fully fasted.^89^

In the setting of labour, a prospective observational study found that 70% and 66% of patients who were admitted to the maternity unit in spontaneous labour or for induction of labour, respectively, had high-risk gastric contents in the first hour on ultrasound, that is fluid >1.5 ml kg^−1^ or the presence of solid food.^82^ Our findings revealed that systemic opioids in the absence of epidural decreased gastric emptying. The reduction in gastric emptying in women who did not have an epidural compared with those who did was likely to be a reflection of the influence of nociceptive stimuli and pain on the activation of the autonomic nervous system and therefore gastroduodenal motility and secretion.^90^^,^^91^ It is probable that the use of epidural opioid in addition to local anaesthetic delays gastric emptying relative to local anaesthetic alone. Over the course of labour, a progressive decrease in solid intake has been described^92^ and the volume of gastric contents has been found to reduce.^38^ In a prospective observational study, 62 patients in labour who had an epidural with local anaesthetic and opioid were allowed to drink clear fluids but not eat any solids and, at the time of full cervical dilatation, 27% of them had high-risk gastric contents on ultrasound, that is fluid >1.5 ml kg^−1^ or the presence of solid food.^93^ The time interval between the insertion of the epidural and gastric ultrasound, possibly owing to an increased cumulative dose of epidural opioid, and the maximum pain score in the last hour of labour, but not the duration of fasting for clear fluids, were risk factors for high-risk gastric contents. These results suggest that clear fluids may be safe in labour. In a randomised controlled trial that compared solids and water in women who were in labour, most of whom received epidural analgesia with local anaesthetic and opioid, the gastric cross-sectional area measured within 1 h of delivery on ultrasound was increased in those who had had solids.^92^ Moreover, in a comparative and prospective observational study, the gastric contents were investigated in 50 patients who were in T3, not in labour, and fasted and 50 women who were in labour, the majority of whom had an epidural, and permitted unrestricted oral intake.^94^ The risk stomach was one in which the volume of fluid was >1.5 ml kg^−1^, fluid was present in the right lateral semirecumbent as well as semirecumbent position or solid food was present and occurred in 80% of patients who were in labour. Importantly, the duration of fasting for solids has been shown to be related to the risk of high-risk gastric contents.^82^ In spontaneous labour and induction of labour, the incidence of high-risk gastric contents on ultrasound was 85–86%, 59–68%, 55–56%, and 48–51% when the women were fasted for <6 h, ≥6 and <8 h, ≥8 and <12 h, or ≥12 h, respectively. In view of this, it should be assumed, in the opinion of the authors, that patients in labour who have eaten solids in the last 8 h still have high-risk contents present in the stomach, and those who have a fasting duration >8 h for solids should be considered for risk stratification by gastric ultrasound if time permits before urgent general anaesthesia. Sufficient time is likely to be available to perform gastric ultrasound in category two but not category one Caesarean delivery. The risk stratification of women with gastric ultrasound may influence decision-making in the situation of category two Caesarean delivery, particularly in relation to the selection of anaesthetic technique and the decision to wake or proceed should failed tracheal intubation occur.^86^ Surprisingly, and to mitigate some of the risk related to solid intake in labour, not all women wish to eat in labour, as underlined by the 29% of those who decided not to have solid intake in a randomised controlled trial.^95^

This narrative review had many limitations. First, most studies were at moderate risk of bias. Deficiencies in the observational studies were found in the domains of confounding, missing data, and selection of the reported result. Problems in the randomised controlled trials were shown in the domains of randomisation process, deviations from the intended interventions, and selection of reported result. Second, heterogeneity was present in the characteristics of the patients and their anaesthetic and obstetric management in the different studies. Populations who tended to be excluded were those with gastro-oesophageal reflux disease, obesity, and diabetes. Differences in anaesthetic management, for example, included the constituents of the epidural solution. Third, inconsistencies were present in the methods used to objectively examine gastric emptying and they all had inherent weaknesses. Gastric ultrasound is user and position dependent. In regard to paracetamol absorption, the plasma concentration is influenced by the volume of distribution and the clearance.^81^ Breath hydrogen analysis provides the orocaecal transit time rather than only the rate of gastric emptying.^77^ Fourth, the variation in results may be due to the quality of observational studies and randomised controlled trials, heterogeneity in the characteristics of the patients and their anaesthetic and obstetric management, and the unpredictability of gastric emptying in pregnancy secondary to multiple factors such as pain. This potential unpredictability highlights the possible preoperative role of gastric ultrasound in women who are pregnant and electively or urgently require general anaesthesia. Fifth, the risk of pulmonary aspiration of gastric contents and the occurrence of respiratory complications is related not only to gastric emptying and thus the presence of high-risk contents in the stomach, but also other factors such as the effect of the gravid uterus on abdominal pressure, progesterone-mediated relaxation of the lower oesophageal sphincter, and reduced gastric pH.^4^

In conclusion, we have been able to summarise and review how gastric emptying varies in the different time periods of pregnancy. Compared with the nonpregnant phase, gastric emptying was decreased in T1 but not T2 and T3. In T3 and before elective Caesarean delivery, the ingestion of carbohydrate drink or tea with milk resulted in no difference in gastric cross-sectional area at 2 h relative to continued fasting or water, and the preoperative ingestion of carbohydrate should be considered in such cases to support enhanced recovery after Caesarean delivery. Compared with the nonpregnant phase and T3, gastric emptying was delayed in labour, although the choice of analgesia had modifying effects. Systemic opioids delayed gastric emptying, epidural analgesia increased it but not back to baseline, epidural analgesia with local anaesthetic and opioid either reduced gastric emptying or made no difference compared with local anaesthetic alone, and intrathecal analgesia delayed it relative to epidural analgesia. The evidence with respect to the postpartum period was conflicting. Overall, the inconsistencies in the literature may reflect the unpredictability of gastric emptying in pregnancy and underline the potential value of gastric ultrasound in women who are pregnant and electively or urgently undergo general anaesthesia.

## Authors’ contributions

Conceptualisation: RH, ND

Search strategy development: RH, ND

Data extraction: JL, RH, ND, JS, CG, PP

Risk of bias: ML, DO

Manuscript writing: JL, RH, ND

Manuscript revision: ND, JL, RH

Supervision: ND

## Declaration of interest

The authors declare that they have no conflicts of interest.
