# Supplementary material for: Gastric emptying in pregnancy and its clinical implications: a narrative review
Source: Br J Anaesth. 2024 Oct 22;134(1):124–67. doi: 10.1016/j.bja.2024.09.005 (PMC11718367; doi:10.1016/j.bja.2024.09.005)
Supplement: Multimedia component 1 [file mmc1.docx]

**Search Strategy**

**CENTRAL**

ID Search Hits

#1 (Gastric NEAR/5 emptying):ti,ab,kw 3848

#2 (Stomach NEAR/5 emptying):ti,ab,kw 1228

#3 (Gastric NEAR/5 content*):ti,ab,kw 844

#4 (Stomach NEAR/5 content*):ti,ab,kw 347

#5 (Transit NEAR/5 time*):ti,ab,kw 1877

#6 MeSH descriptor: [Gastric Emptying] explode all trees 1472

#7 MeSH descriptor: [Gastrointestinal Motility] explode all trees 3036

#8 MeSH descriptor: [Gastrointestinal Transit] explode all trees 603

#9 #1 OR #2 OR #3 OR #4 OR #5 OR #6 OR #7 OR #8 7599

#10 (Caesarean):ti,ab,kw 14897

#11 (Cesarean):ti,ab,kw 14897

#12 (Epidural*):ti,ab,kw 13864

#13 (Labor):ti,ab,kw 17049

#14 (Labour):ti,ab,kw 17033

#15 (Obstetric*):ti,ab,kw 16180

#16 (Parturient*):ti,ab,kw 2606

#17 (Peripartum):ti,ab,kw 341

#18 (Postpartum):ti,ab,kw 11778

#19 (Pregnan*):ti,ab,kw 74796

#20 (Spinal*):ti,ab,kw 30440

#21 MeSH descriptor: [Analgesia, Epidural] explode all trees 2109

#22 MeSH descriptor: [Anesthesia, Epidural] explode all trees 2039

#23 MeSH descriptor: [Anesthesia, Spinal] explode all trees 2511

#24 MeSH descriptor: [Labor Pain] explode all trees 464

#25 MeSH descriptor: [Labor, Obstetric] explode all trees 2524

#26 MeSH descriptor: [Delivery, Obstetric] explode all trees 5732

#27 MeSH descriptor: [Obstetrics] explode all trees 212

#28 MeSH descriptor: [Parturition] explode all trees 548

#29 MeSH descriptor: [Postpartum Period] explode all trees 1914

#30 MeSH descriptor: [Pregnancy] explode all trees 24735

#31 #10 OR #11 OR #12 OR #13 OR #14 OR #15 OR #16 OR #17 OR #18 OR #19 OR #20 OR #21 OR #22 OR #23 OR #24 OR #25 OR #26 OR #27 OR #28 OR #29 OR #30 126145

#32 #9 AND #31 **337**

**CINAHL**

| S26 | S8 AND S25 | **330** |
| --- | --- | --- |
| S25 | S9 OR S10 OR S11 OR S12 OR S13 OR S14 OR S15 OR S16 OR S17 OR S18 OR S19 OR S20 OR S21 OR S22 OR S23 OR S24 | 450,946 |
| S24 | (MH "Pregnancy+") | 237,920 |
| S23 | (MH "Obstetrics") | 6,452 |
| S22 | (MH "Delivery, Obstetric+") | 16,077 |
| S21 | (MH "Labor Pain") | 2,312 |
| S20 | (MH "Anesthesia, Spinal") | 3,601 |
| S19 | (MH "Anesthesia, Epidural") | 3,540 |
| S18 | (MH "Analgesia, Epidural") | 3,943 |
| S17 | Spinal* | 98,924 |
| S16 | Pregnan* | 273,546 |
| S15 | Postpartum | 33,190 |
| S14 | Peripartum | 2,148 |
| S13 | Parturient* | 2,269 |
| S12 | Obstetric* | 62,755 |
| S11 | labor | 60,862 |
| S10 | Epidural* | 15,711 |
| S9 | cesarean | 29,033 |
| S8 | S1 OR S2 OR S3 OR S4 OR S5 OR S6 OR S7 | 7,149 |
| S7 | (MH "Gastrointestinal Motility+") | 3,936 |
| S6 | EXP Gastric Emptying | 0 |
| S5 | Transit N5 time* | 1,828 |
| S4 | Stomach N5 content* | 239 |
| S3 | Gastric N5 content* | 755 |
| S2 | Stomach N5 emptying | 77 |
| S1 | Gastric N5 emptying | 2,012 |

**Embase <1974 to 2022 August 16>**

1 (Gastric adj5 emptying).mp. [mp=title, abstract, heading word, drug trade name, original title, device manufacturer, drug manufacturer, device trade name, keyword heading word, floating subheading word, candidate term word] 20500

2 (Stomach adj5 emptying).mp. [mp=title, abstract, heading word, drug trade name, original title, device manufacturer, drug manufacturer, device trade name, keyword heading word, floating subheading word, candidate term word] 21859

3 (Gastric adj5 content*).mp. [mp=title, abstract, heading word, drug trade name, original title, device manufacturer, drug manufacturer, device trade name, keyword heading word, floating subheading word, candidate term word] 6938

4 (Stomach adj5 content*).mp. [mp=title, abstract, heading word, drug trade name, original title, device manufacturer, drug manufacturer, device trade name, keyword heading word, floating subheading word, candidate term word] 6716

5 (Transit adj5 time*).mp. [mp=title, abstract, heading word, drug trade name, original title, device manufacturer, drug manufacturer, device trade name, keyword heading word, floating subheading word, candidate term word] 22616

6 exp Stomach Emptying/ 21541

7 exp Stomach Motility/ 6007

8 exp Gastrointestinal Motility/ 32879

9 exp Gastrointestinal Transit/ 9917

10 1 or 2 or 3 or 4 or 5 or 6 or 7 or 8 or 9 86373

11 C?esarean.mp. [mp=title, abstract, heading word, drug trade name, original title, device manufacturer, drug manufacturer, device trade name, keyword heading word, floating subheading word, candidate term word] 132177

12 Epidural*.mp. [mp=title, abstract, heading word, drug trade name, original title, device manufacturer, drug manufacturer, device trade name, keyword heading word, floating subheading word, candidate term word] 85001

13 Labo?r.mp. [mp=title, abstract, heading word, drug trade name, original title, device manufacturer, drug manufacturer, device trade name, keyword heading word, floating subheading word, candidate term word] 201187

14 Obstetric*.mp. [mp=title, abstract, heading word, drug trade name, original title, device manufacturer, drug manufacturer, device trade name, keyword heading word, floating subheading word, candidate term word] 202869

15 Parturient*.mp. [mp=title, abstract, heading word, drug trade name, original title, device manufacturer, drug manufacturer, device trade name, keyword heading word, floating subheading word, candidate term word] 10957

16 Peripartum.mp. [mp=title, abstract, heading word, drug trade name, original title, device manufacturer, drug manufacturer, device trade name, keyword heading word, floating subheading word, candidate term word] 9398

17 Postpartum.mp. [mp=title, abstract, heading word, drug trade name, original title, device manufacturer, drug manufacturer, device trade name, keyword heading word, floating subheading word, candidate term word] 89908

18 Pregnan*.mp. [mp=title, abstract, heading word, drug trade name, original title, device manufacturer, drug manufacturer, device trade name, keyword heading word, floating subheading word, candidate term word] 1048336

19 Spinal*.mp. [mp=title, abstract, heading word, drug trade name, original title, device manufacturer, drug manufacturer, device trade name, keyword heading word, floating subheading word, candidate term word] 475272

20 exp Birth/ 29680

21 exp Cesarean Section/ 113602

22 exp Epidural Anesthesia/ 34646

23 exp Labor/ 37833

24 exp Labor Pain/ 3516

25 exp Maternal Care/ 51684

26 exp Obstetrics/ 40810

27 exp Pregnancy/ 733917

28 exp Puerperium/ 74709

29 exp Spinal Anesthesia/ 24875

30 11 or 12 or 13 or 14 or 15 or 16 or 17 or 18 or 19 or 20 or 21 or 22 or 23 or 24 or 25 or 26 or 27 or 28 or 29 1838346

31 10 and 30 **3302**

**Global Health <1973 to 2022 Week 32>**

1 (Gastric adj5 emptying).mp. [mp=abstract, title, original title, heading words, cabicodes words] 3186

2 (Stomach adj5 emptying).mp. [mp=abstract, title, original title, heading words, cabicodes words] 1795

3 (Gastric adj5 content*).mp. [mp=abstract, title, original title, heading words, cabicodes words] 1039

4 (Stomach adj5 content*).mp. [mp=abstract, title, original title, heading words, cabicodes words] 944

5 (Transit adj5 time*).mp. [mp=abstract, title, original title, heading words, cabicodes words] 1946

6 exp Digestive Tract Motility/ 2400

7 exp Stomach Emptying/ 1690

8 exp Stomach Motility/ 480

9 exp Transit Time/ 909

10 1 or 2 or 3 or 4 or 5 or 6 or 7 or 8 or 9 8501

11 C?esarean.mp. [mp=abstract, title, original title, heading words, cabicodes words] 10223

12 Epidural*.mp. [mp=abstract, title, original title, heading words, cabicodes words] 1367

13 Labo?r.mp. [mp=abstract, title, original title, heading words, cabicodes words] 28227

14 Obstetric*.mp. [mp=abstract, title, original title, heading words, cabicodes words] 16857

15 Parturient*.mp. [mp=abstract, title, original title, heading words, cabicodes words] 1143

16 Peripartum.mp. [mp=abstract, title, original title, heading words, cabicodes words] 820

17 Postpartum.mp. [mp=abstract, title, original title, heading words, cabicodes words] 14449

18 Pregnan*.mp. [mp=abstract, title, original title, heading words, cabicodes words] 138516

19 Spinal*.mp. [mp=abstract, title, original title, heading words, cabicodes words] 11466

20 exp Caesarean Section/ 6495

21 exp Maternity/ 737

22 exp Obstetrics/ 5363

23 exp Parturition/ 3540

24 exp Postpartum Period/ 6253

25 exp Pregnancy/ 109044

26 exp Puerperium/ 1618

27 11 or 12 or 13 or 14 or 15 or 16 or 17 or 18 or 19 or 20 or 21 or 22 or 23 or 24 or 25 or 26 186212

28 10 and 27 **124**

**Ovid MEDLINE(R) ALL <1946 to August 17, 2022>**

1 (Gastric adj5 emptying).mp. [mp=title, book title, abstract, original title, name of substance word, subject heading word, floating sub-heading word, keyword heading word, organism supplementary concept word, protocol supplementary concept word, rare disease supplementary concept word, unique identifier, synonyms] 16586

2 (Stomach adj5 emptying).mp. [mp=title, book title, abstract, original title, name of substance word, subject heading word, floating sub-heading word, keyword heading word, organism supplementary concept word, protocol supplementary concept word, rare disease supplementary concept word, unique identifier, synonyms] 819

3 (Gastric adj5 content*).mp. [mp=title, book title, abstract, original title, name of substance word, subject heading word, floating sub-heading word, keyword heading word, organism supplementary concept word, protocol supplementary concept word, rare disease supplementary concept word, unique identifier, synonyms] 5396

4 (Stomach adj5 content*).mp. [mp=title, book title, abstract, original title, name of substance word, subject heading word, floating sub-heading word, keyword heading word, organism supplementary concept word, protocol supplementary concept word, rare disease supplementary concept word, unique identifier, synonyms] 3100

5 (Transit adj5 time*).mp. [mp=title, book title, abstract, original title, name of substance word, subject heading word, floating sub-heading word, keyword heading word, organism supplementary concept word, protocol supplementary concept word, rare disease supplementary concept word, unique identifier, synonyms] 13663

6 exp Gastric Emptying/ 10630

7 exp Gastrointestinal Motility/ 38430

8 exp Gastrointestinal Transit/ 5146

9 1 or 2 or 3 or 4 or 5 or 6 or 7 or 8 61505

10 C?esarean.mp. [mp=title, book title, abstract, original title, name of substance word, subject heading word, floating sub-heading word, keyword heading word, organism supplementary concept word, protocol supplementary concept word, rare disease supplementary concept word, unique identifier, synonyms] 82254

11 Epidural*.mp. [mp=title, book title, abstract, original title, name of substance word, subject heading word, floating sub-heading word, keyword heading word, organism supplementary concept word, protocol supplementary concept word, rare disease supplementary concept word, unique identifier, synonyms] 54883

12 Labo?r.mp. [mp=title, book title, abstract, original title, name of substance word, subject heading word, floating sub-heading word, keyword heading word, organism supplementary concept word, protocol supplementary concept word, rare disease supplementary concept word, unique identifier, synonyms] 159634

13 Obstetric*.mp. [mp=title, book title, abstract, original title, name of substance word, subject heading word, floating sub-heading word, keyword heading word, organism supplementary concept word, protocol supplementary concept word, rare disease supplementary concept word, unique identifier, synonyms] 200450

14 Parturient*.mp. [mp=title, book title, abstract, original title, name of substance word, subject heading word, floating sub-heading word, keyword heading word, organism supplementary concept word, protocol supplementary concept word, rare disease supplementary concept word, unique identifier, synonyms] 8692

15 Peripartum.mp. [mp=title, book title, abstract, original title, name of substance word, subject heading word, floating sub-heading word, keyword heading word, organism supplementary concept word, protocol supplementary concept word, rare disease supplementary concept word, unique identifier, synonyms] 6376

16 Postpartum.mp. [mp=title, book title, abstract, original title, name of substance word, subject heading word, floating sub-heading word, keyword heading word, organism supplementary concept word, protocol supplementary concept word, rare disease supplementary concept word, unique identifier, synonyms] 81309

17 Pregnan*.mp. [mp=title, book title, abstract, original title, name of substance word, subject heading word, floating sub-heading word, keyword heading word, organism supplementary concept word, protocol supplementary concept word, rare disease supplementary concept word, unique identifier, synonyms] 1096062

18 Spinal*.mp. [mp=title, book title, abstract, original title, name of substance word, subject heading word, floating sub-heading word, keyword heading word, organism supplementary concept word, protocol supplementary concept word, rare disease supplementary concept word, unique identifier, synonyms] 432706

19 exp Analgesia, Epidural/ 8842

20 exp Anesthesia, Epidural/ 14043

21 exp Anesthesia, Spinal/ 12910

22 exp Labor Pain/ 1319

23 exp Labor, Obstetric/ 48108

24 exp Delivery, Obstetric/ 88574

25 exp Obstetrics/ 24170

26 exp Parturition/ 20597

27 exp Postpartum Period/ 72847

28 exp Pregnancy/ 976622

29 10 or 11 or 12 or 13 or 14 or 15 or 16 or 17 or 18 or 19 or 20 or 21 or 22 or 23 or 24 or 25 or 26 or 27 or 28 1699026

30 9 and 29 **2089**

**Scopus**


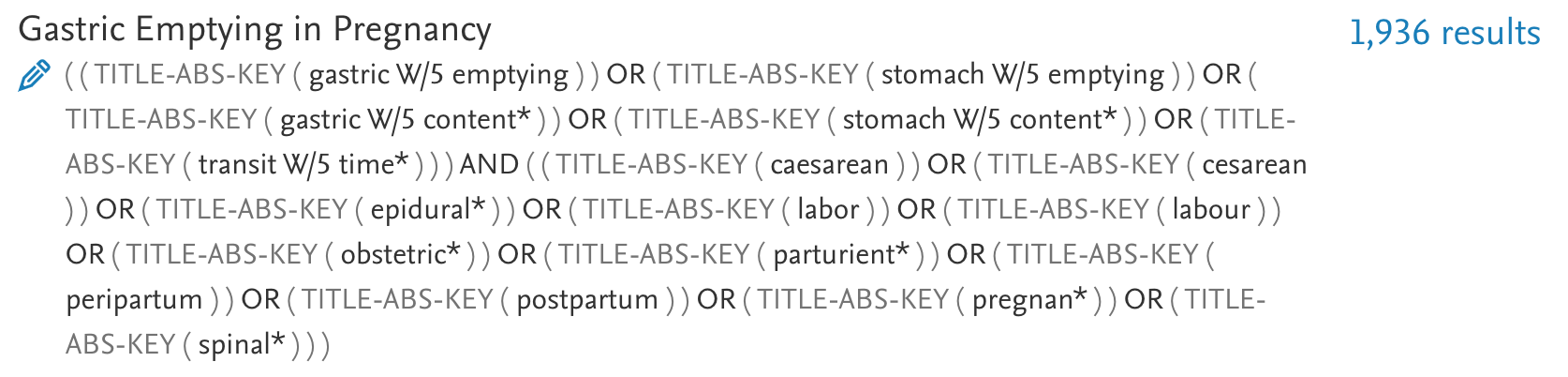


**Web of Science Core Collection**

# Searches:

1: TS=(Gastric NEAR/5 emptying) Date run: Thu Aug 18 2022 16:11:34 GMT+0100 (British Summer Time) Results: 15808

2: TS=(Stomach NEAR/5 emptying) Date run: Thu Aug 18 2022 16:11:51 GMT+0100 (British Summer Time) Results: 2144

3: TS=(Gastric NEAR/5 content*) Date run: Thu Aug 18 2022 16:14:25 GMT+0100 (British Summer Time) Results: 4406

4: TS=(Stomach NEAR/5 content*) Date run: Thu Aug 18 2022 16:15:03 GMT+0100 (British Summer Time) Results: 8197

5: TS=(Transit NEAR/5 time*) Date run: Thu Aug 18 2022 16:15:20 GMT+0100 (British Summer Time) Results: 24667

6: #5 OR #4 OR #3 OR #2 OR #1 Date run: Thu Aug 18 2022 16:15:28 GMT+0100 (British Summer Time) Results: 52731

7: TS=(C$esarean) Date run: Thu Aug 18 2022 16:15:42 GMT+0100 (British Summer Time) Results: 64702

8: TS=(Epidural*) Date run: Thu Aug 18 2022 16:15:53 GMT+0100 (British Summer Time) Results: 48166

9: TS=(Labo$r) Date run: Thu Aug 18 2022 16:16:07 GMT+0100 (British Summer Time) Results: 334347

10: TS=(Obstetric*) Date run: Thu Aug 18 2022 16:16:21 GMT+0100 (British Summer Time) Results: 101262

11: TS=(Parturient*) Date run: Thu Aug 18 2022 16:16:29 GMT+0100 (British Summer Time) Results: 7829

12: TS=(Peripartum) Date run: Thu Aug 18 2022 16:16:37 GMT+0100 (British Summer Time) Results: 6978

13: TS=(Postpartum) Date run: Thu Aug 18 2022 16:17:36 GMT+0100 (British Summer Time) Results: 72483

14: TS=(Pregnan*) Date run: Thu Aug 18 2022 16:17:44 GMT+0100 (British Summer Time) Results: 597728

15: TS=(Spinal*) Date run: Thu Aug 18 2022 16:17:52 GMT+0100 (British Summer Time) Results: 339571

16: #7 OR #8 OR #9 OR #10 OR #11 OR #12 OR #13 OR #14 OR #15 Date run: Thu Aug 18 2022 16:18:20 GMT+0100 (British Summer Time) Results: 1365967

17: #6 AND #16 Date run: Thu Aug 18 2022 16:18:29 GMT+0100 (British Summer Time) **Results: 956**
